# Economic and epidemiological evaluation of interventions to reduce the burden of hepatitis C in Yunnan province, China

**DOI:** 10.1371/journal.pone.0245288

**Published:** 2021-01-13

**Authors:** Alastair Heffernan, Yanling Ma, Shevanthi Nayagam, Polin Chan, Zhongdan Chen, Graham S. Cooke, Yan Guo, Chuntao Liu, Mark Thursz, Wanyue Zhang, Xiaobing Zhang, Xiujie Zhang, Manhong Jia, Timothy B. Hallett

**Affiliations:** 1 School of Public Health, Imperial College London, London, United Kingdom; 2 Yunnan Center for Disease Control and Prevention, Kunming, China; 3 World Health Organization Western Pacific Regional Office, Manila, Philippines; University of Waterloo, CANADA

## Abstract

**Background:**

The paradigm shift in hepatitis C virus (HCV) treatment options in the last five years has raised the prospect of eliminating the disease as a global health threat. This will require a step-change in the number being treated with the new direct-acting antivirals (DAAs). Given constrained budgets and competing priorities, policy makers need information on how to scale-up access to HCV treatment. To inform such decisions, we examined the cost effectiveness of screening and treatment interventions in Yunnan, China.

**Methods and findings:**

We simulated the HCV epidemic using a previously published model of HCV transmission and disease progression, calibrated to Yunnan data, and implemented a range of treatment and screening interventions from 2019. We incorporated treatment, diagnosis, and medical costs (expressed in 2019 US Dollars, USD) to estimate the lifetime benefits and costs of interventions. Using this model, we asked: is introducing DAAs cost effective from a healthcare sector perspective; what is the optimal combination of screening interventions; and what is the societal return on investment of intervention? The incremental cost-effectiveness ratio (ICER) of switching to DAAs with a median cost of 7,400 USD (50,000 Chinese Yuan) per course is 500 USD/disability adjusted life year (DALY) averted; at a threshold of 50% of Yunnan gross domestic product (2,600 USD), switching to DAAs is cost effective 94% of the time. At this threshold, the optimal, cost-effective intervention comprises screening people who inject drugs, those in HIV care, men who have sex with men, and ensuring access to DAAs for all those newly diagnosed with HCV. For each USD invested in this intervention, there is an additional 0·80 USD (95% credible interval: 0·17–1·91) returned through reduced costs of disease or increased productivity. Returns on investment are lower (and potentially negative) if a sufficiently long-term horizon, encompassing the full stream of future benefits, is not adopted. The study had two key limitations: costing data were not always specific to Yunnan province but were taken from China-level studies; and modelled interventions may require more operational research to ensure they can be effectively and efficiently rolled-out to the entire province.

**Conclusions:**

Introducing DAAs is cost effective, the optimal package of screening measures is focussed on higher risk groups, and there are likely to be positive returns from investing in such HCV interventions. Our analysis shows that targeted investment in HCV interventions will have net benefits to society; these benefits will only increase as DAA costs fall.

## Introduction

The key to tackling the hepatitis C epidemic is the implementation of intervention strategies that increase the number diagnosed and treated with direct-acting antivirals (DAAs) [1]. These revolutionary drugs offer greatly improved cure rates over the previous standard of care along with fewer side effects [2–4].

Despite their promise, it is unclear how DAA access can be improved or whether it will be cost effective to do so [5]. Past studies have quantified the impact of introducing DAAs in the context of patients already in care [6–9]. Other studies have investigated the cost effectiveness or return on investment of increasing the number treated, but these studies have not utilised dynamic models; they cannot, therefore, incorporate the full benefits of intervention including the effects of averting infections nor can they capture the impacts of diminishing returns of screening as prevalence in the population as a whole declines [10–12]. No study that we are aware of has made an economic case for introducing DAAs and scaling-up screening in a dynamic model at the level of the whole population. These are critical considerations to take into account when appraising expanding treatment access across a region.

This study will help to address these questions in the specific case of Yunnan province, China. China as a whole is home to the largest number of people infected with hepatitis C virus (HCV) in the world [13, 14]. Recent modelling work has demonstrated that if China does not make progress in implementing interventions to tackle the hepatitis C epidemic then global elimination targets will not be met until decades after the 2030 target [1]. Yunnan province itself has a large population of people who inject drugs (PWID) [15, 16]. possibly due to Yunnan’s position along key heroin trafficking routes into China [17, 18]. HCV has primarily been studied among PWID in the province, among whom anti-HCV prevalence is higher than among PWID in the rest of China [19]. Yunnan has been at the forefront of efforts in China to combat infectious disease: the province established some of the first opioid substitution therapy centres and needle and syringe programmes in the country [20–22] and has taken the lead in promoting safe sex among female sex workers (FSW) [20]. Non-governmental organisations (NGOs) play a role in delivering public health-interventions including to FSW, men who have sex with men (MSM), PWID and pregnant women [23].

The provincial Center for Disease Control and Prevention is now planning to scale up interventions designed to reduce the burden of HCV. This analysis is designed to inform this intervention scale up by answering the following three research questions:

- Is introducing DAAs cost effective from a healthcare sector perspective?
- If DAAs were to be introduced, what is the optimal set of interventions to roll them out?
- What is the societal return on investment for implementing the recommended HCV intervention package?

## Methods

### Overview

To answer these questions, we adapted a previously published mathematical model [1] to simulate the epidemiology of HCV in Yunnan. A list of the equations defining the model and associated parameters is given in the S1 File. To the epidemiological model we added an economic model to make projections of the costs and benefits of interventions and used this to assess the cost effectiveness of introducing DAAs; to identify the optimal mix of intervention elements once DAAs are introduced; and to calculate the return on investment (ROI) of the recommended intervention package from a societal perspective as described below.

### Modelling approach

#### Model structure

**Fig 1:**
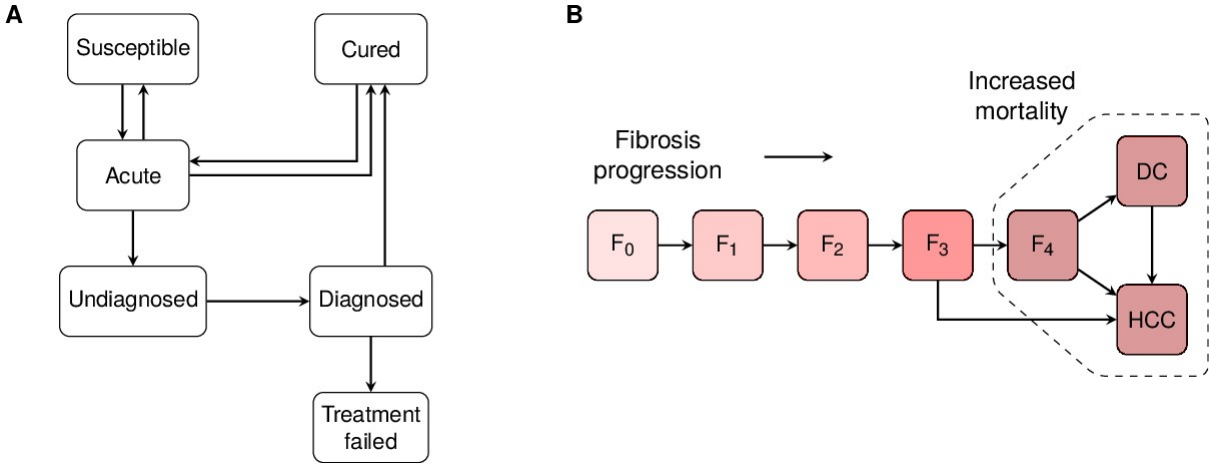
Schematic of mathematical model. Boxes represent compartments of the model while arrows denote transitions that may depend upon age, sex, risk group, or duration of infection and can vary over time. Everyone is in one compartment of the cascade of care (A). These compartments are further subdivided by age, sex, and risk group. Infection results in people entering the natural history model (B): hepatitis C virus disease progresses through five METAVIR [24] fibrosis stages. The potential impacts of age and male sex on progression and mortality rates are accounted for in calibration. Those in the cured compartment of (A) have reduced or zero disease progression rates depending on disease stage. DC = decompensated cirrhosis. HCC = hepatocellular carcinoma.

The model dynamically simulates the population of Yunnan, grouped according to infection and treatment status. The rate at which HCV infection occurs is governed by age, time, and population-specific calibrated transmission risks. The process of diagnosis and treatment initiation and resultant cure are simulated. Reinfection following cure is also modelled (Fig 1A). Unless cured, disease progression occurs according to a natural history model parameterised by literature review (Fig 1B) with increased HCV-related mortality from stages: compensated cirrhosis (F4), decompensated cirrhosis, and hepatocellular carcinoma (HCC).

The modelled population is stratified by age, sex, and risk group: MSM, PWID, FSW, and the remaining population termed the general population. PWID are categorised as either being registered (those reachable by health interventions and risk reduction initiatives, see below) or unregistered according to a recent survey in Yunnan [15]. Background age, sex, and time-specific mortality is simulated and PWID experience an additional mortality risk related to, amongst other things, risk of over-dose [25].

#### Risk group structure

The risk of infection varies by group and is calibrated according to the procedure described below. All susceptible compartments experience a force of infection that is the product of an infection risk multiplied by an appropriate prevalence. The general population force of infection is the product of the overall population prevalence and the general population risk of infection. This risk is calibrated by fitting flexible splines that vary by both age and time.

The higher-risk population groups (PWID, MSM and FSW) experience this general population force of infection plus an additional force of infection equal to the prevalence in that group multiplied by a risk group-specific and time-specific risk of infection. This additional risk of infection (one for each of PWID, MSM and FSW) is fit in calibration.

The choice of risk groups ((PWID, MSM and FSW) was motivated by observed higher prevalence values in these groups. By fitting risks in the way done here, no statement is being made about the precise modality of infection; in particular, higher risk in FSW can be due to other risk behaviours than sex work. But considering FSW as a distinct group is of value here from an operational standpoint as they will be reached by interventions that are distinct from MSM, PWID and so on.

#### Historical treatment and harm reduction coverage

Access to harm reduction interventions among registered PWID is scaled up from 20% to 70% between 2007 and 2011 (held constant thereafter) reducing risk of HCV infection in those reached by the intervention by 75% [26]. Historical treatment is assumed to consist of the former standard of care (pegylated interferon plus ribavirin) only. In 2015 to 2016, 10,000 people were diagnosed. Of these, 15% are estimated to have completed treatment (30% were offered treatment of whom 50% completed treatment courses) according to local data. The same numbers are assumed to have been diagnosed and subsequently treated in 2017 and 2018.

We introduce DAAs in a range of scenarios (see below). We assume an appropriate DAA combination is given to the patient and apply appropriate values for proportion achieving sustained viral response based on the literature (see the list of parameters in the S1 File for further details).

#### Calibration

The model is calibrated in a Bayesian framework [27] to Yunnan-specific data on anti-HCV prevalence by risk group (PWID, FSW, MSM, and pregnant women) obtained through sentinel surveys (S1 File) and numbers of deaths by age, sex, and type (HCC or decompensated cirrhosis) from cause of death monitoring systems in Yunnan. The calibration procedure draws 1,000 samples from the parameter posteriors which are then used to produce forecasts of quantities of interest. Outputs are reported as medians and associated 95% credible intervals (2·5 and 97·5 percentiles). For data to which the model are fit see S1 File.

#### Scenarios

We defined a status quo scenario in which all calibrated risk and intervention coverages and effectiveness assumptions are held constant from 2019 onwards. We defined a first intervention scenario in which DAAs are adopted in place of pegylated interferon plus ribavirin (denoted scenario A) but with no other changes in diagnosis or treatment coverage.

We then defined a set of intervention scenarios (Table 1). These were considered attainable and practical within Yunnan and rely on harnessing several different methods of reaching people for HCV testing as a means of scaling up the proportion diagnosed and subsequently treated in the province.

**Table 1:** Yunnan intervention scenario details.

Scenario	Name	Details
SQ	Status quo	• 20% (2007) rising to 70% (2011) coverage of harm reduction among registered PWID.
		• pegylated interferon plus ribavirin only treatment available.
		• 10,000 diagnosed 2015–2018; constant rate of diagnosis thereafter.
		• 15% of new HCV diagnoses complete treatment from 2015 onwards.
A	Switch to DAAs	Treatment is with DAAs from 2019 onwards; diagnosis and treatment rates unchanged from status quo.
B	Treatment (DAAs) for majority newly diagnosed	80% of those newly diagnosed positive with HCV treated regardless of disease stage (except where contraindicated)
C	Screen MSM	7,000 MSM offered HCV test annually 2019–2030, through engagement with relevant NGOs. 90% offered HCV test accept. 90% of those testing HCV positive complete treatment.
D	Screen FSW	20,000 FSW offered HCV test annually 2019–2030, through engagement with relevant NGOs. 90% offered HCV test accept. 90% of those testing HCV positive complete treatment.
E	Screen PWID	20,000 registered PWID offered HCV test annually 2019–2030, through engagement with relevant NGOs. 90% offered HCV test accept. 90% of those testing HCV positive complete treatment.
F	Screen in HIV care	70,000 HIV-positive people offered HCV test annually 2019–2030. 90% offered HCV test accept. 90% of those testing HCV positive complete treatment.
G	Screen pregnant women	Offer all pregnant women HCV test during one antenatal care appointment from 2019–2030. 90% of pregnant women accept HCV test. 90% of those testing HCV positive complete treatment.
H	Screen 40 year olds	10% of 40 year olds are offered screening each year from 2019–2030. 90% offered HCV test accept. 90% of those testing HCV positive complete treatment.

PWID = people who inject drugs. HCV = hepatitis C virus. DAAs = direct-acting antivirals. MSM = men who have sex with men. FSW = female sex worker. NGOs = non-governmental organisations. Screening interventions make the assumption that 90% of those offered screening accept it, and of those testing positive, 90% accept treatment. The numbers that can be reached in screening programmes (scenarios C-H) are based on local estimates. All features of status quo (expect where explicitly changed) are retained in the other intervention scenarios. All intervention scenarios A-H ‘switch off’ after 2030. The colour of each row corresponds to the colours used for each scenario in the results’ figures, see Figs 5 and 6.

#### Costing model

**Fig 2:**
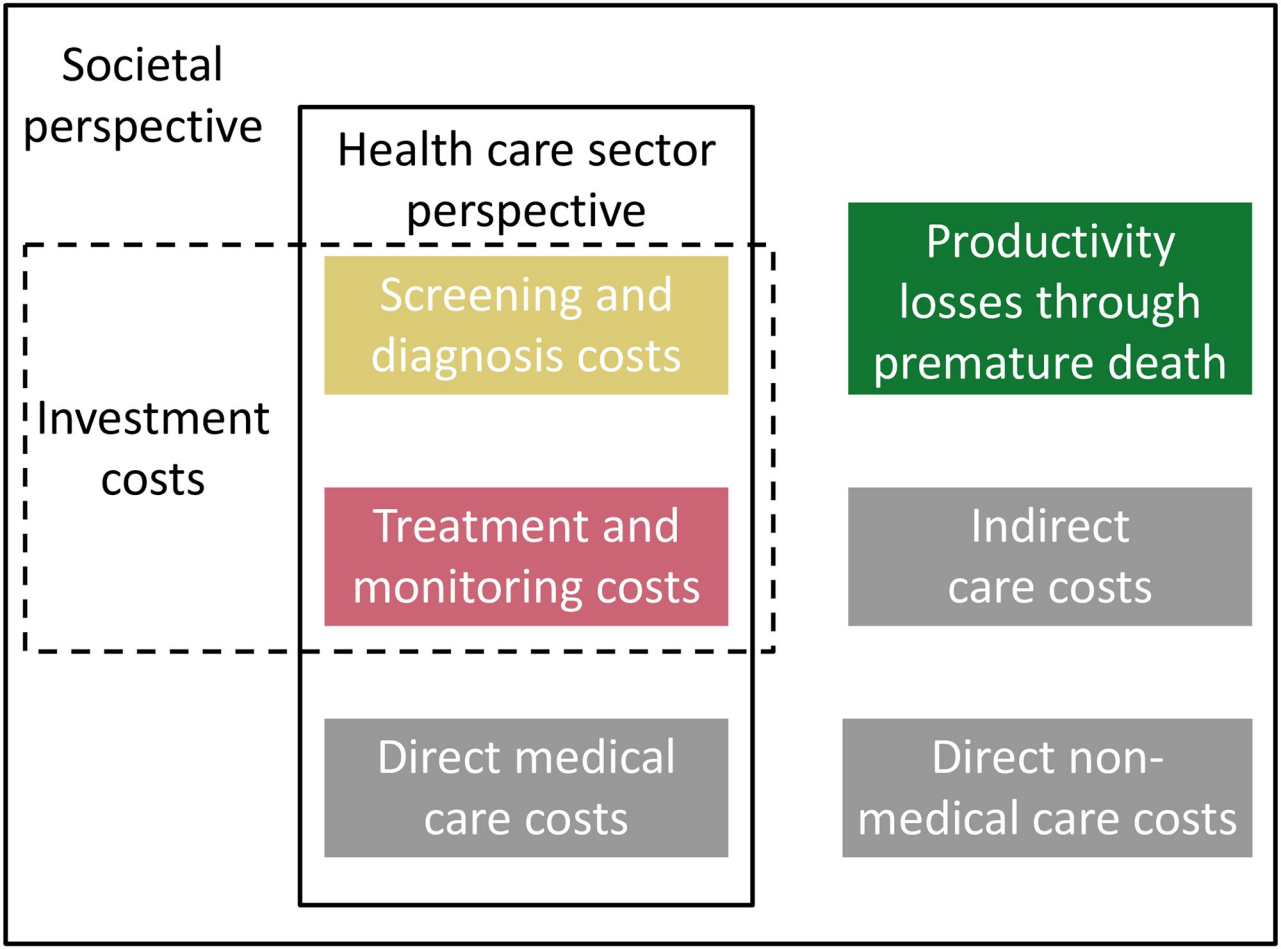
Costing inventory. Colours refer to the cost category; the grey set of boxes are collectively referred to as the “care costs”. The components of each cost are given in Table 2 where colour indicates to which category they belong. Investment costs refer to the categorisation of costs used in the return on investment analysis, see below.

We developed an economic model to estimate the relevant total costs associated with HCV infection. Costs are presented in 2019 US Dollars (USD), converted from Chinese Yuan (CNY) at a rate of 1 USD = 6.75 CNY [28]. We categorised costs as shown in Fig 2; unit costs are given in Table 2. The model distinguishes healthcare sector perspective costs (screening and diagnosis, treatment and monitoring, and direct medical care costs); intervention investment costs (screening and diagnosis, and treatment and monitoring costs); and societal perspective costs (all cost categories). When assessing cost effectiveness, we utilise only the healthcare perspective costs; when estimating the return on investment we consider the change in all costs (a societal perspective) relative to the required intervention investment costs (see analysis section below). In keeping with the National Institute for Health and Care Excellence guidelines, we exclude medical costs unrelated to HCV [29]; the effect of including these would be to reduce the costs averted through intervention by accounting for the additional medical expenditure incurred in added life years [30].

**Table 2:** Costing data.

Cost	Value/range (distribution)	Justification and references
Cost of anti-HCV test (unit cost)	3 USD (Uniform: ±20%)	Local stakeholders
Cost of HCV-RNA test (unit cost)	42 USD (Uniform: ±20%)	Local stakeholders; sum HCV-RNA (22 USD) and genotype test (20 USD)
Cost of diagnostic work up	52 USD (Uniform: ±20%)	Cost of diagnostic work up for HBV less HBV-specific tests (S. Nayagam, personal communication)
Cost of course of DAA treatment	5,900–8,900 USD (Uniform)	Local stakeholders
Cost of course of pegylated interferon plus ribavirin treatment	5,000 USD (Uniform: ±20%)	Local stakeholders
Cost of treatment monitoring per week	25 USD (Uniform: ±20%)	Literature [6]
Cost of care for patients in care stages F0-F3	410 USD (Lognormal: 95% CI 0·5-2x)	Direct medical: 120 USD (local stakeholders)
		Direct non-medical: 180 USD [33]
		Indirect: 120 USD [33]
Cost of care for patients in care stages F4	1,200 USD (Lognormal: 95% CI 0·5-2x)	Direct medical: 740 USD (local stakeholders)
		Direct non-medical: 210 USD [33]
		Indirect: 240 USD [33]
Cost of care for patients in care stages DC	2,700 USD (Lognormal: 95% CI 0·5-2x)	Direct medical: 1,300 USD (midpoint of data from local stakeholders)
		Direct non-medical: 800 USD [33]
		Indirect: 580 USD
Cost of care for patients in care stages HCC	5,700 USD (Lognormal: 95% CI 0·5-2x)	Direct medical: 3,600 USD (midpoint of data from local stakeholders)
		Direct non-medical: 1,500 USD [33]
		Indirect: 700 USD [33]
Multiplicative change in care costs following SVR	0 –stages F0 to F3	Literature [7, 34]
	0·709 (Lognormal: 95% CI 0·592–0·855)–all other stages	
Average cost of year of working life lost	2,600 USD (Uniform: ±20%))	Local stakeholders; original value adjusted for assumption of 4% unemployment [35]

HCV = hepatitis C virus. HBV = hepatitis B virus. DAA = direct-acting antiviral. USD = United States Dollars. DC = decompensated cirrhosis. HCC = hepatocellular carcinoma. SVR = sustained viral response. Ranges shown indicate the range of values explored in the primary analyses. Colours indicate to which group of costs the parameter belongs, see Fig 2. Values are converted from Chinese Yuan (CNY) at rate of 1 USD = 6.75 CNY. Direct medical, direct non-medical and indirect care costs may not sum to the total value due to rounding of values (after converting currencies) in the table; values in the model are in CNY and sum correctly.

The model calculates the difference in cost in a year between two scenarios and sums these differences, discounted at 3% per year. Interventions are started in 2019 and ‘turned off’ in in 2030 but the model is run until 2100 (approximating a lifetime horizon) in order that the full streams of costs and benefits are captured [31, 32]. Horizon length and discount rate are varied in sensitivity analyses.

#### Health outcomes

Disability-adjusted life years (DALYs) were used to evaluate net health benefits upon implementation of the different intervention scenarios (S1 File). The health impact of interventions is calculated by summing the difference in DALYs year by year between two scenarios, discounted at 3% per year, to a horizon year of 2100. The impact of varying disability weights between the ranges reported by the Global Burden of Disease Group is assessed.

### Answering the research questions

#### Is introducing DAAs cost effective from a healthcare sector perspective?

Cost effectiveness is assessed by calculating the mean change in costs incurred in the health system (using only the healthcare perspective costs, see Fig 2) and the mean change in DALYs after introduction of DAAs (Scenario A) compared to status quo and calculating the incremental cost-effectiveness ratio (ICER) [36]:

$$ICER=\frac{\Delta cost}{\Delta DALY}.$$

An ICER value below the cost-effectiveness threshold signifies that the health benefit from introducing DAAs exceeds the health opportunity cost. We adopt the threshold of 0·5x per capita gross domestic product (GDP) / DALY averted [37]–equal to 2,600 USD in Yunnan [38]. Sensitivity of our conclusions to changes in DAA and care costs is explored.

#### If DAAs were to be introduced, what is the optimal set of interventions to roll them out?

We simulated all combinations of the remaining interventions, which assume that DAAs are available (B-H see Table 1) and constructed the cost-effectiveness frontier. This describes the optimum combination of interventions to scale-up access to DAA treatment at a given cost-effectiveness threshold. Uncertainty in whether a particular combination strategy is cost effective is assessed by constructing the cost-effectiveness acceptability frontier (S1 File) [39].

#### What is the societal return on investment for implementing the recommended HCV intervention package?

The ROI is calculated for this optimal package of interventions as the benefits (the overall change in societal perspective costs) divided by the intervention investment costs (defined in Fig 2). The ROI was evaluated across a range of DAA and health care costs.

## Results

### The HCV epidemic in Yunnan without DAAs

**Fig 3:**
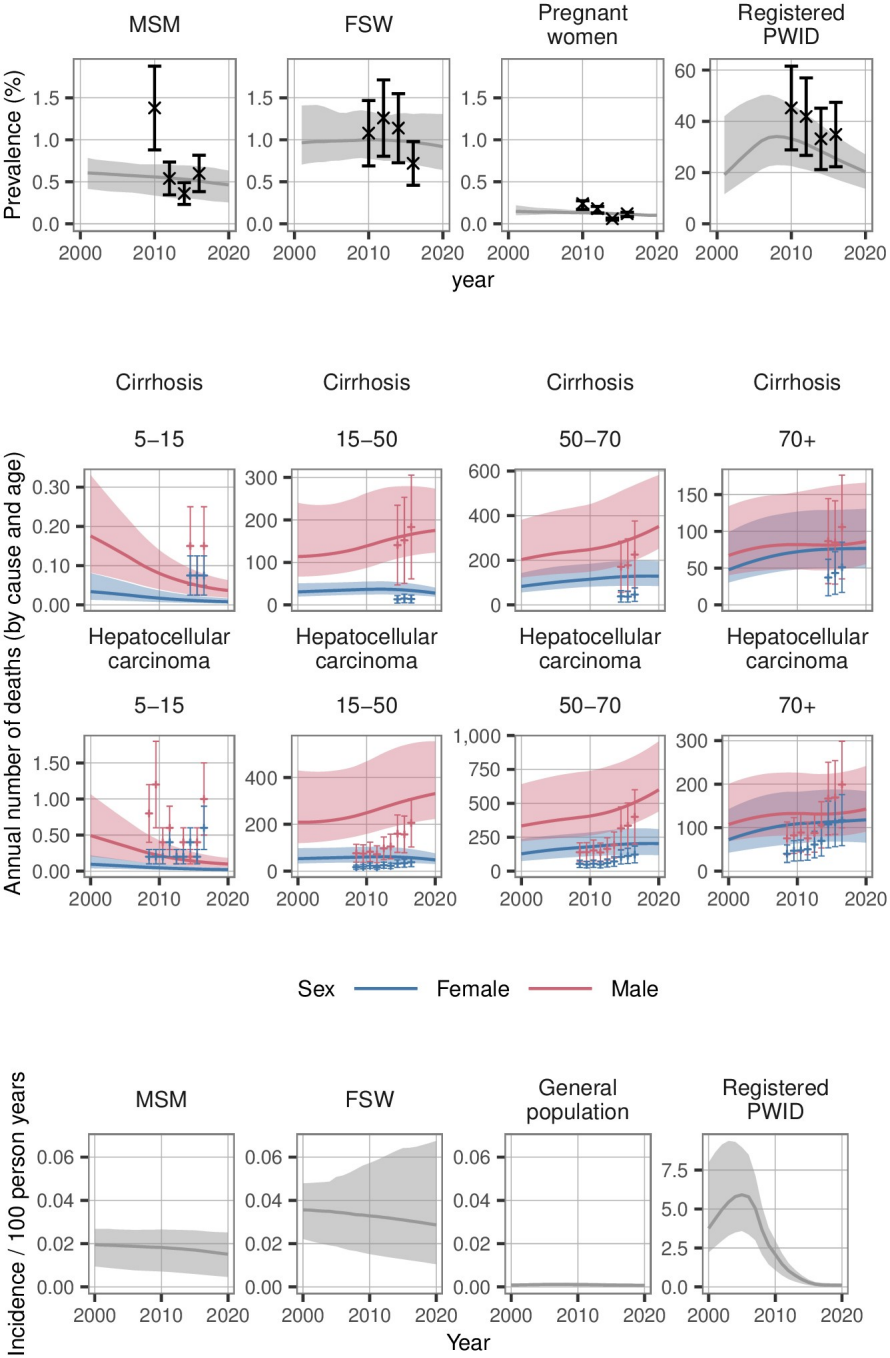
Calibration results. Shown are results of the calibrations procedure to (A) prevalence data by risk group and (B) mortality data by sex and age. MSM = men who have sex with men. FSW = female sex workers. PWID = people who inject drugs.

The results of the calibrated model are shown in Fig 3. In 2018, incidence rates overall were estimated to be 1·4 per 100,000 susceptible per year (95% CrI: 0·6–2·5). Incidence rate varies considerably by risk group: PWID—570 per 100,000 per year (220–900); MSM– 17 per 100,000 per year (6–26); and FSW– 30 per 100,000 per year (12–65). The population sizes of these risk groups in 2018 are 190,000 (PWID), 125,000 (MSM), and 27,000 (FSW). There have been modest reductions in prevalence among FSW and greater reduction among registered PWID in recent years.

Maintaining status quo intervention coverage and retaining pegylated interferon plus ribavirin as the only treatment option results in 28,000 deaths between 2019 and 2030, while there would are an estimated 6,060 new infections over the same period. By 2030 there are 103,000 active HCV infections (95% CrI: 87,000–121,000) in Yunnan in status quo.

### Is introducing DAAs cost effective from a healthcare sector perspective?

**Fig 4:**
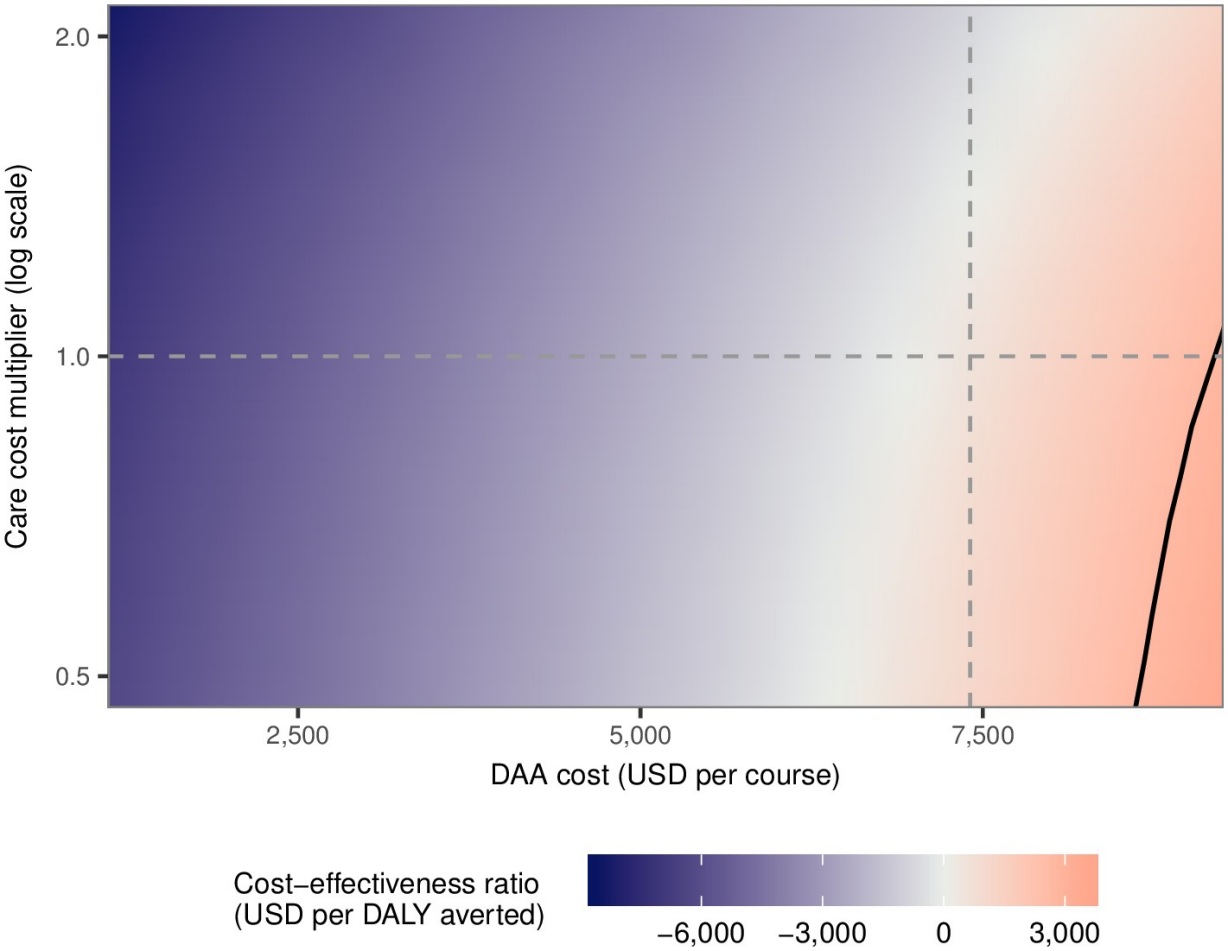
Cost effectiveness of introducing DAAs upon varying care and DAA costs. The black separatrix indicates the divide between cost-effective (above the line) and not cost-effective (below the line) simulations at a cost-effectiveness threshold of 2,600 USD (line drawn where the mean incremental cost-effectiveness ratio is cost effective). The dashed vertical and horizontal grey lines indicate the costs of direct-acting antivirals and care cost multiplier used in the primary analysis. DAA = direct-acting antiviral. USD = United States Dollars.

We estimated the mean ICER for switching from pegylated interferon plus ribavirin to DAAs to be 500 USD/DALY averted. If the cost of DAAs is 7,400 USD (in local terms: 50,000 CNY), in the middle of the 5,900–8,900 USD range considered in the analysis, then the percentage of runs that would be classified as being cost effective at the assumed threshold of 50% of Yunnan GDP (2,600 USD) is 94%. The ICER decreases with lower DAA costs or higher cost of treating those with HCV disease (Fig 4 and S1 File). If the cost of DAAs is at the bottom of the range we considered (5,500 USD) then more than 99% of runs are classified as cost saving. The variation of the ICER to changes in methodological parameters (horizon length, discount rate, and DALY disability weights) is shown in S1 File.

Scenario A averts 460 deaths by 2030 (95% CrI: 390–560) and 73 new infections (25–141). Introducing DAAs slightly reduces the number with active viraemic infection to 100,000 (85,000–118,000) in 2030, down from 103,000 in status quo.

### If DAAs were to be introduced, what is the optimal set of interventions to roll them out?

**Fig 5:**
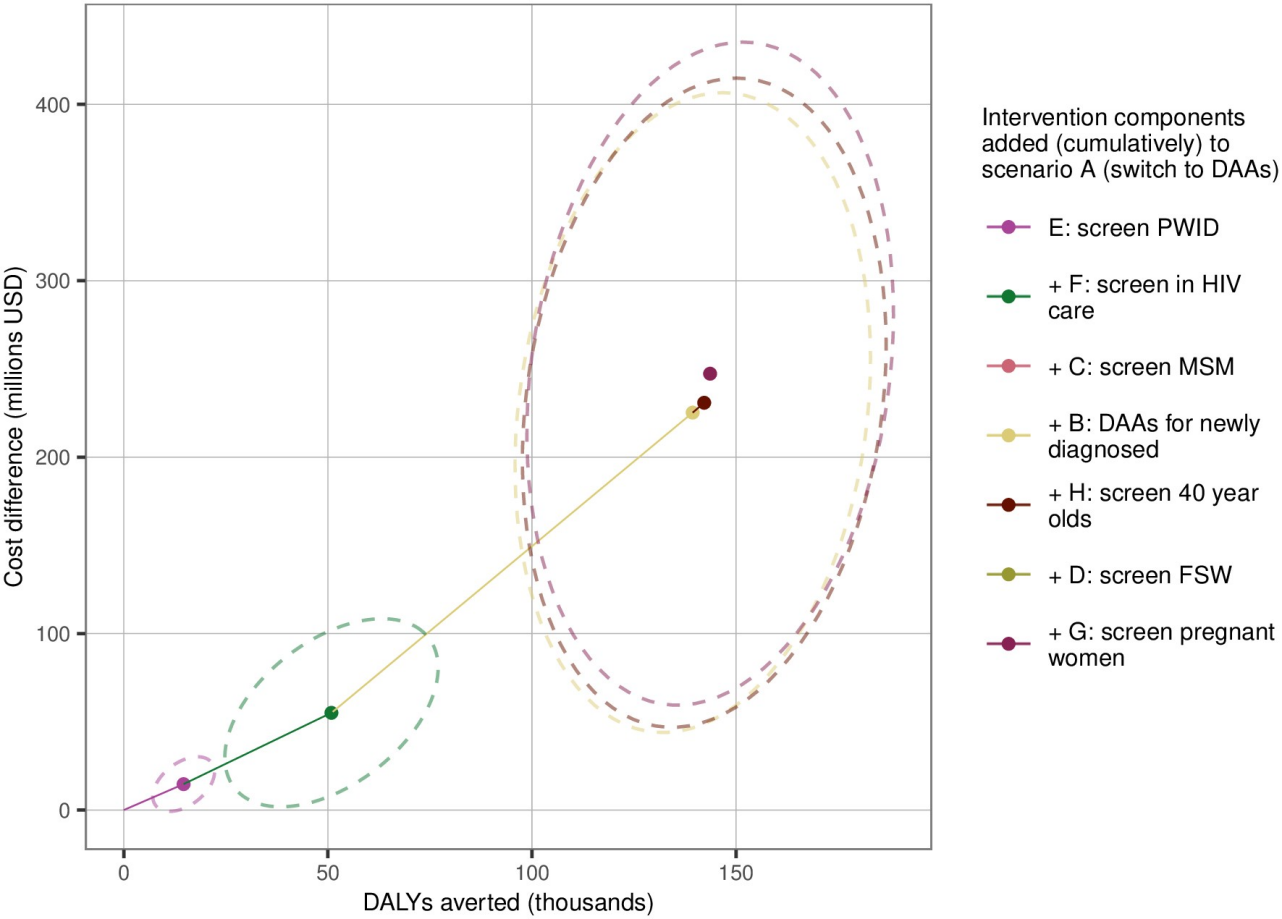
Cost-effectiveness frontier from health care sector perspective. Shown are the mean DALYs averted versus cost differences between each successive intervention and the previous (coloured dots); the first scenario is compared to status quo. The slope of the coloured connecting lines indicates the mean ICERs relating each intervention to the next most cost-effective intervention; connecting lines are not shown for those interventions that are not cost effective at a threshold of 2,600 USD/DALY averted. Ellipses show spread of cost-benefit pairs around the mean. In order, the visible ellipses, mean cost-effect points and lines indicating the ICER are (moving from bottom left to upper right): + E, + F, +B, +H, then +G. Scenarios C and D cannot be seen on the figure as they have minimal impact relative to the other scenarios considered; the mean values and ellipses are covered by the previous scenario components added (F and H respectively)–which are the colours shown since these drive the health and cost changes seen on the figure. See Fig 6 for further clarification. USD = United States Dollars. DALYs = disability-adjusted life years. PWID = people who inject drugs. MSM = men who have sex with men. DAAs = direct-acting antivirals. FSW = female sex workers.

Fig 5 shows the order in which subsequent intervention elements would be added after introducing DAAs (scenario A) according to the cost-effectiveness analysis (ICERs, cost changes, DALYs averted and the additional annual costs associated with each intervention component are shown by scenario in S1 File). The order of intervention elements added to the optimal strategy (and their associated ICERs) is: + PWID screening (scenario E, ICER = US$1,000 / DALY averted); + screening in HIV care (F, $1,100); + screen MSM (C, $1,700); + DAAs for newly diagnosed (B, $1,900); and + 40 year-old age cohort screening (H, $2,000). This combined set of interventions is cost-effective in 66% of simulations at a threshold of 2,600 USD, as shown on the cost-effectiveness acceptability frontier; this figure also shows the optimal set of interventions across a range of cost-effectiveness thresholds and their associated probability of being cost-effective (S1 File).

**Fig 6:**
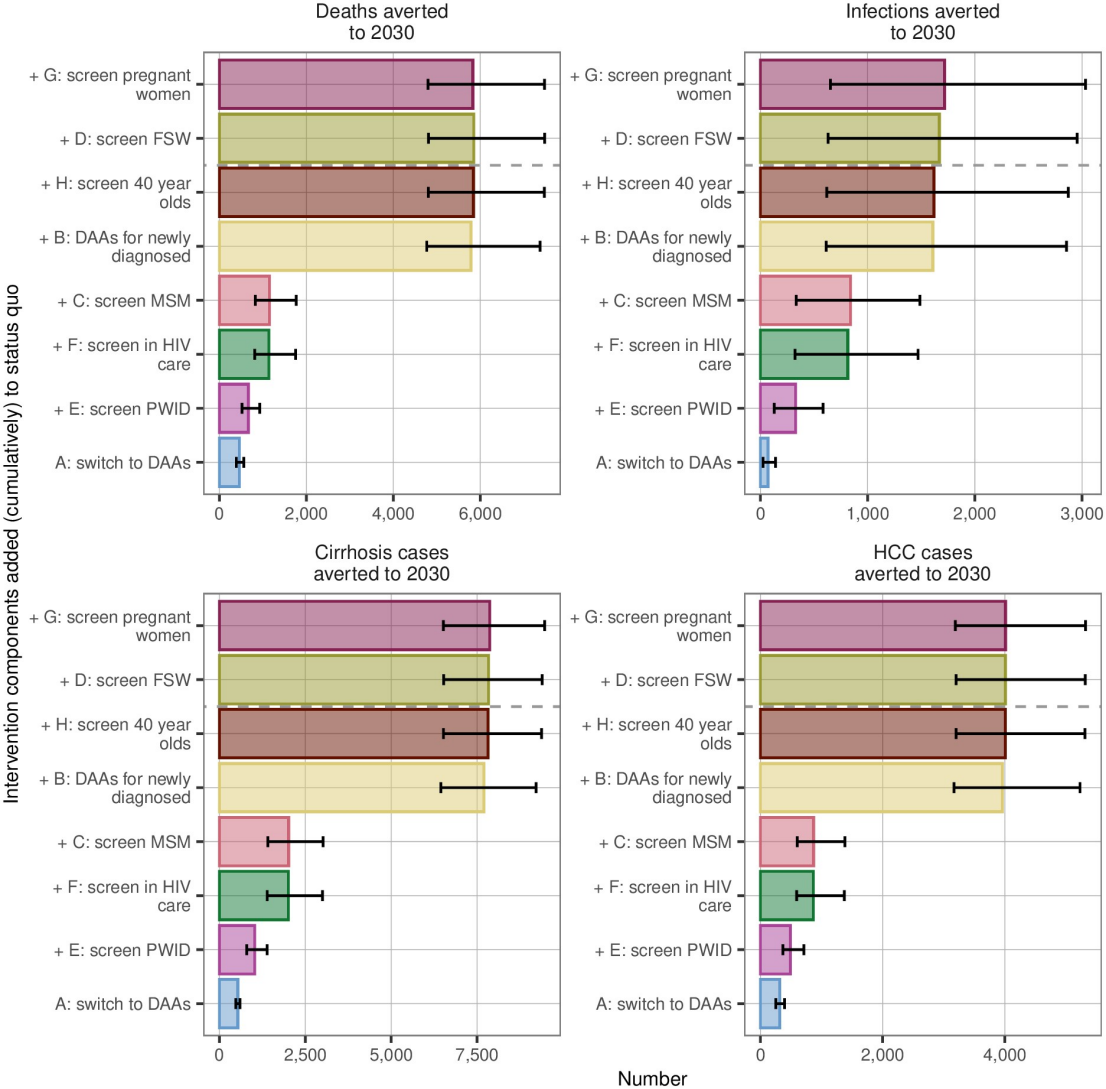
Cumulative deaths, infections, and end-stage liver disease sequelae averted. Shown are the cumulative changes in numbers of deaths, infections, cases of cirrhosis (stages F4 and decompensated cirrhosis), and HCC averted 2019–2030 due to each intervention element. The dashed grey line indicates where adding interventions is no longer cost effective; the optimum package comprises all intervention components below the dashed line. FSW = female sex workers. DAAs = direct-acting antivirals. MSM = men who have sex with men. PWID = people who inject drugs. HCC = hepatocellular carcinoma.

The cumulative impact on deaths and infections averted by 2030 due to each intervention component is illustrated in Fig 6. The optimal set of interventions (E, F, C, B, and H) results in 5,800 deaths averted (95% CrI: 4,800–7,500), 1,600 infections averted (600–2,900), and a reduction in active infections by 2030 to 55,000 (47,000–69,000). For projections of optimal intervention impact over time see S1 File.

### What is the societal return on investment for implementing the recommended HCV intervention package?

**Fig 7:**
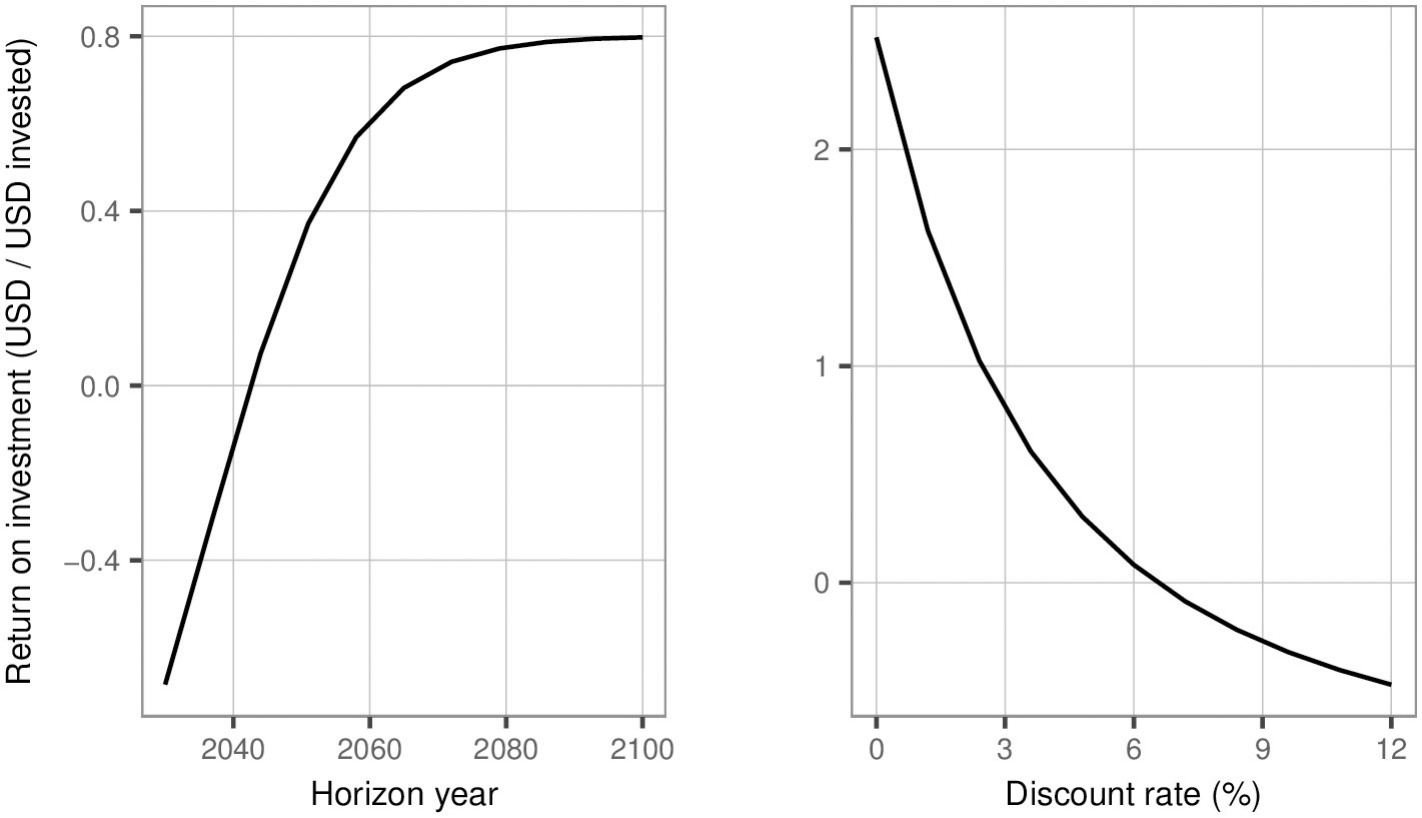
Sensitivity of median return on investment to changes in horizon year and discount rate. Upon varying horizon year, the baseline value for the discount rate (3%) was applied; upon varying discount rate, the baseline value of horizon year (lifetime horizon– 2100) was applied. USD = United States Dollars.

Implementation of the optimal package of interventions results in a lifetime, discounted return of 4.8 billion USD (95% CrI: 2·53–8·13 billion USD) on an investment of 2.7 billion USD (95% CrI: 2·53–8·13 billion USD). This corresponds to an ROI of 0·80 USD/USD invested (95% CrI: 0·17–1·91) when considered over a lifetime horizon (to 2100). The impact of changing horizon year or discount rate on the ROI is shown in Fig 7. Break-even points exist at which the ROI changes from negative to positive, specifically at a horizon year of less than 2042 or at a discount rate of over 6·5%.

In sensitivity analysis, if DAA costs were assumed to be over 7,400 USD and care costs less than half the primary analysis assumption then only 23% of runs involved returns less than investment (negative ROI); the average ROI of this subset of runs was still positive (0·13 USD/USD invested). If, on the other hand, DAA costs are reduced to 2,200 USD the ROI exceeds 10. ROI increases still further as DAA costs decrease beyond this or health care costs associated with HCV are higher than expected (S1 File).

We explored the extent to which ROI varied by parameter (S1 File). Two parameters were responsible for the majority of the variation: care costs (increasing care costs increases the ROI) and the difference between DAA and PEG-IFN+RBV costs (increasing the cost of DAAs relative to PEG-IFN+RBV reduces the ROI). Correspondingly, the key lever to improving the returns on this investment that can plausibly be varied is the cost of DAAs; diagnosis costs (the other cost that could also potentially be pushed down) has a negligible impact on ROI.

## Discussion

### Summary of findings

We have addressed three question of high priority for Yunnan, China—and many other places—as they develop plans to scale up access to new HCV treatment. In short, we have found that: introducing DAAs—even at current high costs—is cost effective; testing higher risk group individuals for HCV through existing intervention programmes is the best way of scaling up screening; and the optimal strategy delivers benefits to society of 1.8 USD for every 1 USD invested. These questions are addressed in more detail in turn.

#### Is introducing DAAs cost effective from a healthcare sector perspective?

Introducing DAAs into the current care context of Yunnan province is expected to be cost effective at a threshold of 2,600 USD/DALY averted. Low treatment and diagnosis rates render this a relatively ineffective intervention, however, with only 460 deaths averted by 2030 and a reduction in number of active infections of less than 3%.

#### If DAAs were to be introduced, what is the optimal set of interventions to roll them out?

Having a major epidemiological impact requires scaling up interventions that increase access to DAAs. The optimal cost-effective package of interventions comprises screening registered PWID accessible through harm reduction programmes, HIV-infected individuals presenting for routine care, MSM engaged via non-governmental organisations (NGOs), offering DAAs for all as they are diagnosed, and offering 40 year-old age cohort screening. By 2030, this combined package would avert 5,800 deaths and 1,600 new infections while reducing the number actively infected by approximately half compared to status quo.

#### What is the societal return on investment for implementing the recommended HCV intervention package?

The optimal strategy as defined above offers a return on investment into screening and treatment of 80%; this is because the marginal extra cost of DAAs and screening is being returned in the form of increased productivity (through increased health of the workforce) and through defrayed costs of care for persons with liver disease. Reductions in DAA costs to 2,200 USD (approximately 15,000 CNY), a value closer to that observed in other upper-middle-income countries like South Africa [40], can drive this societal return to over 1,000%. Such gains require a long-term view to be taken: reducing the horizon year, or heavily discounting future costs and benefits, results in negative ROI (as shown by the presence of break-even points upon varying these quantities). This is because near-term costs to diagnose and cure individuals are only offset by benefits (such as averted deaths or health complications of those who would otherwise have been infected) that manifest decades in the future.

### Strengths and limitations

To produce an economic and epidemiological analysis of HCV interventions in Yunnan, we have had to manage various limitations. A common issue when modelling key populations is the reliability of estimates of population size, particularly for PWID which is the most important population in this model. This is partially mitigated here as we incorporated numbers that explicitly aim to estimate the total number of PWID, including those who are unregistered PWID and would not normally be counted among the PWID accessing NGO or other programmes [15]. We have also had to assume that HCV prevalence and risk behaviours among unregistered PWID are the same as observed among registered PWID. Similarly, cirrhosis and HCC mortality estimates do not provide information on aetiology. HCV-attributable mortality estimates are based on surveys of the proportion of cirrhosis and HCC cases that are attributable to HCV in China. Broad ranges for HCV-attributable mortality were chosen to mitigate this limitation. In short, the prevalence and mortality estimates adopted reflect the best available data, while the approach taken in calibration was designed to explore a range of scenarios to improve the robustness of the results to possible uncertainty in calibration data.

With regards the economic modelling component, direct medical care costs available for Yunnan itself were utilised, however, estimates made for these costs for other regions have been shown to be heterogeneous and are often based on limited empirical data [6, 7]. To account for this, we sampled care costs in our primary analysis over a wide range of values (0·5-2x base costs). To quantify direct non-medical and indirect medical costs, we have had to make use of estimates pertaining to hepatitis B virus disease from ten years ago [33]. The limitations of such data are managed in a number of ways: firstly, these costs are not included in the cost-effectiveness analysis (as they are not healthcare sector perspective costs) and so do not affect results to research questions 1 and 2 that only utilised healthcare sector perspective data. Secondly, in the societal perspective, these costs are smaller than both direct medical costs and the impacts of lost productivity and so will play an undersized role in shaping the results. Lastly, we varied costs over wide ranges to ensure results incorporate a significant degree of uncertainty in cost estimates.

There is of course the further question over the feasibility of the intervention scenarios simulated. Scenario B involves treating 80% of all those newly testing HCV positive in the province, regardless of disease stage. While clearly a challenging and ambitious target, strategies have recently been adopted in countries as diverse as Egypt and Australia to improve DAA coverage among newly diagnosed individuals. Approaches include empowering healthcare professionals outside of tertiary care centres to prescribe and monitor DAA treatment courses [40, 41]. In this way, loss to follow up experienced between the various stages of testing and treatment could be minimised and an 80% treatment target for those being diagnosed could become a plausible goal.

Several other intervention components involve outreach screening designed to make use of existing networks. There are likely to be some costs incurred in the “piggy backing” design of the interventions by the NGOs and health sector agencies responsible for delivering HCV tests (either as initial overheads or through the need to contribute to running costs). We neglected these, since the very small contribution screening and diagnosis as a whole make to the overall costs (result not shown), and the minimal impact varying these costs has on results (S1 File), suggests that only an exceptionally large increase in delivery costs would alter the conclusions drawn here. The point remains, however, that more operational research may be required to minimise the costs incurred through delivering HCV screening via other channels and to ensure that these strategies can reach as many people per year as simulated here.

Past work evaluating the introduction of DAAs in China has calculated the cost effectiveness of switching to DAAs within cohorts of already-diagnosed individuals. Chen et al. (2016) investigated the cost effectiveness of providing DAAs to genotype 1b patients in China from a healthcare sector perspective [7]. The high costs of DAAs (96,000 USD in that study) prevented the intervention being cost effective. Chen and Chen (2017), Liu et al. (2018), and Lu et al. (2018) all performed similar analyses, though the progressively lower DAA prices (12,000 USD, 8,500 USD, and 4,400 USD respectively) led to the conclusion in all three papers that providing DAAs in place of pegylated interferon plus ribavirin is likely to be cost effective [6, 8, 9]. We also find switching to DAAs to be cost effective, with a probability of 94% at a price of 7,400 USD (within the range of past work). But our work goes beyond previous analyses by utilising a dynamic simulation of a whole population and not just a cohort already in care. This allowed us generate a recommended option for improving access to treatment in a way not done in previous research, make projections of costs at the level of the whole population and not a subset, and to build in secondary benefits (such as morbidity averted through prevented infections) that other studies have neglected.

## Conclusions

This analysis has shown, firstly, that introducing DAAs in Yunnan is cost effective at current prices and likely to be cost saving if prices decrease further towards DAA costs reached in other countries [41]. Secondly, we have derived a recommended set of intervention components to improve access to DAAs that is cost effective once a decision is made to adopt DAAs. This cost-effective package of interventions incorporates screening of high-risk groups, which averts future infections, and a change of approach to ongoing diagnosis such that people are offered DAA treatment immediately upon testing HCV positive. Thirdly, this analysis has shown that, from a societal perspective, investing in screening for HCV and subsequent DAA treatment courses offers a positive return on investment; this return will only increase as DAA costs decrease. This societal return on investment can be used by policy makers to assess whether or not to invest in HCV interventions and should inform ongoing discussions concerning treatment scale up in Yunnan province and beyond.

## Supporting information

S1 File Supplementary appendix. Additional methods, results and figures.
(DOCX)

S1 Data Spreadsheet containing all data in the figures. (XLSX)

## Abbreviations

CNY: Chinese Yuan
DAAs: direct-acting antivirals
DALY: disability adjusted life year
FSW: female sex worker
HCC: hepatocellular carcinoma
HCV: hepatitis C virus
ICER: incremental cost-effectiveness ratio
MSM: men who have sex with men
PWID: people who inject drugs
ROI: return on investment
