# Supplementary material for: Economic and epidemiological evaluation of interventions to reduce the burden of hepatitis C in Yunnan province, China
Source: PLoS One. 2021 Jan 13;16(1):e0245288. doi: 10.1371/journal.pone.0245288 (PMC7806158; doi:10.1371/journal.pone.0245288)
Supplement: S1 File — Additional methods, results and figures. (DOCX) [file pone.0245288.s001.docx]

S1 File: Economic and epidemiological evaluation of interventions to reduce the burden of hepatitis C in Yunnan, China

Alastair Heffernan^1^, Yanling Ma^2^, Shevanthi Nayagam^1^, Polin Chan^3^, Zhongdan Chen^3^, Graham S. Cooke^1^, Yan Guo^2^, Chuntao Liu^2^, Mark Thursz^1^, Wanyue Zhang^2^, Xiaobing Zhang^2^, Xiujie Zhang^2^, Manhong Jia^2*^, Timothy B. Hallett^1*^

**1** School of Public Health, Imperial College London, United Kingdom, **2** Yunnan Center for Disease Control and Prevention, Kunming, China, **3** World Health Organization Western Pacific Regional Office, Manila, Philippines

^*^ [jiamanhong@yncdc.cn](mailto:jiamanhong@yncdc.cn), [timothy.hallett@imperial.ac.uk](mailto:timothy.hallett@imperial.ac.uk)

Table of Contents

[Supplementary methods 1](#_Toc32768926)

[Supplementary input data 3](#_Toc32768927)

[Supplementary methods tables 4](#_Toc32768928)

[Supplementary results tables 10](#_Toc32768929)

[References 16](#_Toc32768930)

## Supplementary methods

*Details of epidemiological modelling*

The epidemiological model utilised in this paper is derived from the model used in a previous publication.^1^ Here we briefly describe the model as it is applied to the case of Yunnan. For detailed discussion of aspects of the model unchanged in this analysis, please refer to the web appendix of the original paper.^1^

A model was constructed to make projections of the hepatitis C epidemic in Yunnan and to analyse the impact of a set of intervention packages. To simulate the full course of the hepatitis C epidemic, a mathematical model was constructed that incorporates: population dynamics (birth, natural death and migration); age and sex stratification; dynamic infection with hepatitis C virus (HCV); differential risk of infection among the simulated risk groups – people who inject drugs (PWID), female sex workers (FSW) and men who have sex with men (MSM); disease progression leading to cirrhosis; increased hepatitis C specific mortality due to complications following onset of cirrhosis; historical rates of diagnostic screening; historical rates of HCV treatment and PWID risk-reduction interventions; treatment success and treatment failure; reduced rates of disease progression and mortality following treatment success; and possible reinfection following cure. The model is calibrated to HCV viraemic prevalence in PWID, FSW, MSM and pregnant women (see Supplementary Input Data below) and HCV-attributable mortality estimates by age and sex. Historical and future demographic information, HCV genotype distributions, PWID data, and past coverage of harm reduction and HCV interventions are used as model inputs. Uncertainty is accounted for in a statistical framework allowing disease burden projections to be made by sampling from calibrated parameter posterior distributions using the incremental mixture importance sampling method described here.^2^

The differential equations underpinning the mathematical model and a full list of parameters is given in the section Supplementary Methods Tables below.

*Disability weights*

Disability-adjusted life years (DALYs) are calculated as the sum of the difference in years of life lost (YLLs) and years lived with disability (YLDs) between scenarios.^3^ YLLs per year are calculated as the total number of people who would be alive were it not for premature hepatitis C virus (HCV) death. YLDs per year are calculated by multiplying the disability weights (0 for fibrosis stages F0 to F4, 0·178 for decompensated cirrhosis, and 0·452 for hepatocellular carcinoma, HCC) by the average annual number with each sequela. The disability weight for persons with HCC is assumed to be the same between cured and non-cured persons but the disability weight for persons with decompensated cirrhosis is assumed reduced to zero (the same as for compensated cirrhosis) following cure. The difference in DALYs between scenario and comparator is denoted the $\Delta DALY$, where a reduction in DALYs in a scenario relative to a comparator results in a positive $\Delta DALY$.

*Construction of efficiency frontier*

To find the optimal set of screening and treatment scale-up interventions after introducing DAAs, an iterative approach is taken: interventions B-H are individually simulated above a baseline of scenario A. The ICERs for each intervention (B to H) are compared and the intervention with the lowest ICER is selected for inclusion in the optimal intervention set. This strategy, comprising scenario A plus the new intervention component, forms the new baseline. The process is then repeated: the remaining interventions are simulated above this new baseline and the intervention component with the lowest ICER is added to the optimal set up until no additional interventions are cost effective (at a given cost-effectiveness threshold). Uncertainty in the choice of intervention strategy at a range of cost-effectiveness threshold values is assessed by constructing the cost-effectiveness acceptability frontier, see the following section.

*Construction of cost-effectiveness acceptability frontier*

Th cost-effectiveness acceptability frontier (CEAF) is constructed according to the method laid out here.^4^ In brief, the net benefit of an intervention is defined – at a given cost-effectiveness threshold $\lambda$ – as: $\lambda\Delta cost-\Delta DALY$. The model is run across a range of economic and epidemiological values. For a given run of the model, the net benefit is calculated for each intervention and that intervention with the highest net benefit (provided it is positive – indicating the intervention is cost-effective at the given $\lambda$) is identified. This process is repeated many times and for a range of values of $\lambda$. The proportion of runs in which an intervention offers the greatest net-benefit can then be plotted (these correspond to cost-effectiveness acceptability curves - CEACs).

By calculating the mean costs and mean benefits, the mean net benefit can be calculated for a range of values of $\lambda$ by intervention. The intervention with the highest expected net benefit for a given $\lambda$ is the optimal decision at that threshold.^5^ This optimal intervention is illustrated on the cost-effectiveness acceptability curves to indicate the cost-effectiveness frontier – this is achieved by drawing the CEACs as dashed lines and, where an intervention is optimal, by drawing the CEAF as a solid line, see Figure 3. The value of $\lambda$ at which an intervention becomes the optimal choice corresponds to that interventions ICER; for a list of values see Table 4.

## Supplementary input data

*Population sizes*

Where no reference is given – data are informed by the records of Yunnan CDC.

- People who inject drugs - PWID:
  - percentage of people who inject drugs among 15-64 year old population: 0·57% (range: 0·53-0·61%);^6^
  - percentage women among PWID: 9·0%;^6^
  - percentage PWID main drug injected opioids: 99·9%;^7^
  - percentage PWID HIV+: 16·2%; and
  - percentage PWID registered: 14·2%.^6^
- Men who have sex with men - MSM:
  - percentage men MSM: 0·5%;
  - percentage MSM HIV+: 7·1%;
- Female sex workers - FSW:
  - percentage women FSW: 0·11% - based off of estimated 26,000 FSW in 2016 divided by overall number women that year.
- General population:
  - proportion HIV+: 0·26%.

*Calibration data*

Below are tables containing data to which the model is fit.

**Table 1 - Anti-HCV prevalence data.** HCV = hepatitis C virus. FSW = female sex worker. MSM = men who have sex with men. PWID = people who inject drugs. Data provided by Yunnan Centres for Disease Control are given in the table below. These were converted to viraemic (HCV-RNA) prevalence rates according to a China-specific viraemic rate of 60%.^8^ Confidence intervals for high-risk groups were imputed from anti-HCV prevalence among people who inject drugs in China as a whole;^7^ confidence intervals for pregnant women were imputed from anti-HCV prevalence among the overall population in China as a whole.^8^

| **Group / Year** | **2010** | **2012** | **2014** | **2016** |
| --- | --- | --- | --- | --- |
| **FSW** | 1·8 | 2·1 | 1·9 | 1·2 |
| **MSM** | 2·3 | 0·9 | 0·6 | 1 |
| **Pregnant Women** | 0·4 | 0·3 | 0·1 | 0·2 |
| **PWID** | 75·4 | 69·8 | 55·3 | 58·1 |

**Table 2 – Total number of deaths due to cirrhosis.** Hepatitis C virus-attributable cirrhosis mortality was estimated to be 5-25% (central value: 15%) of the total number of cirrhosis deaths shown in the table.^9–12^

| **Year / Age / Sex** | | **5-15** | **15-50** | **50-70** | **70+** |
| --- | --- | --- | --- | --- | --- |
| **2014** | **Female** | 0 | 88 | 255 | 248 |
|  | **Male** | 1 | 938 | 1138 | 578 |
| **2015** | **Female** | 0 | 100 | 248 | 289 |
|  | **Male** | 0 | 1013 | 1184 | 566 |
| **2016** | **Female** | 0 | 92 | 308 | 341 |
|  | **Male** | 1 | 1222 | 1503 | 706 |

**Table 3 – Total number of deaths due to hepatocellular carcinoma.** Hepatitis C virus-attributable hepatocellular carcinoma (HCC) mortality was estimated to be 10-30% (central value: 20%) of the total number of HCC deaths shown in the table.^13,14^

| **Year / Age / Sex** | | **5-15** | **15-50** | **50-70** | **70+** |
| --- | --- | --- | --- | --- | --- |
| **2008** | **Female** | 1 | 85 | 269 | 200 |
|  | **Male** | 4 | 381 | 697 | 377 |
| **2009** | **Female** | 0 | 94 | 241 | 234 |
|  | **Male** | 6 | 375 | 699 | 410 |
| **2010** | **Female** | 1 | 116 | 274 | 239 |
|  | **Male** | 2 | 413 | 764 | 443 |
| **2011** | **Female** | 2 | 81 | 246 | 251 |
|  | **Male** | 3 | 361 | 689 | 377 |
| **2012** | **Female** | 1 | 126 | 298 | 305 |
|  | **Male** | 1 | 486 | 819 | 437 |
| **2013** | **Female** | 1 | 113 | 356 | 347 |
|  | **Male** | 2 | 526 | 962 | 532 |
| **2014** | **Female** | 2 | 161 | 521 | 537 |
|  | **Male** | 0 | 799 | 1577 | 834 |
| **2015** | **Female** | 0 | 169 | 559 | 562 |
|  | **Male** | 2 | 784 | 1676 | 847 |
| **2016** | **Female** | 3 | 190 | 612 | 586 |
|  | **Male** | 5 | 1029 | 2000 | 995 |

## Supplementary methods tables

*Partial differential equations defining the model*

$$\frac{\partial S}{\partial t}+\frac{\partial S}{\partial a}=\left( 1-p_{inf.} \right)b\left( t \right)+\sum_{g} \lambda_{i}^{clear}A-\left( \Lambda_{il}\left( t \right)+\mu_{ijl}^{nat.}\left( t \right)-\nu\left( t \right) \right)S$$

$$\frac{\partial A}{\partial t}+\frac{\partial A}{\partial a}=p_{inf.}b\left( t \right)+\Lambda_{il}\left( t \right)S-\left( \lambda_{i}^{clear}+\lambda_{i}^{chronic}+\mu_{ijl}^{nat.}\left( t \right)-\nu\left( t \right) \right)A$$

$$\frac{\partial A^{k}}{\partial t}+\frac{\partial A^{k}}{\partial a}+ \frac{\partial A^{k}}{\partial d}=\theta_{reinf}\Lambda_{il}\left( t \right)C^{k}+\sum_{k^{'}\to k} \lambda_{ij}^{k^{'}\to k}A^{k^{'}} -\left( \lambda_{i}^{clear}+\lambda_{i}^{chronic}+\sum_{k\to k^{''}} \lambda_{ij}^{k\to k^{''}} +\mu_{ijd}^{k}+\mu_{ijl}^{nat.}\left( t \right)-\nu\left( t \right) \right)A^{k}$$

$$\frac{\partial U^{k}}{\partial t}+\frac{\partial U^{k}}{\partial a}+ \frac{\partial U^{k}}{\partial d}=\lambda_{i}^{chronic}\left( A^{k}+\delta_{k,0}A \right)+\sum_{k^{'}\to k} \lambda_{ij}^{k^{'}\to k}U^{k^{'}}-\left( \delta^{k}\left( t \right)+\sum_{k\to k^{''}} \lambda_{ij}^{k\to k^{''}} +\mu_{ijd}^{k}+\mu_{ijl}^{nat.}\left( t \right)-\nu\left( t \right) \right)U^{k}$$

$$\frac{\partial D^{k}}{\partial t}+\frac{\partial D^{k}}{\partial a}+ \frac{\partial D^{k}}{\partial d}=\delta^{k}\left( t \right)U^{k}+\sum_{k^{'}\to k} \lambda_{ij}^{k^{'}\to k}D^{k^{'}}-\left( \tau^{k}\left( t \right)+\sum_{k\to k^{''}} \lambda_{ij}^{k\to k^{''}} +\mu_{ijd}^{k}+\mu_{ijl}^{nat.}\left( t \right)-\nu\left( t \right) \right)D^{k}$$

$$\frac{\partial T^{k}}{\partial t}+\frac{\partial T^{k}}{\partial a}+ \frac{\partial T^{k}}{\partial d}=\tau^{k}\left( t \right)D^{k}+\sum_{k^{'}\to k} \lambda_{ij}^{k^{'}\to k}T^{k^{'}}-\left( \phi_{g}+\sum_{k\to k^{''}} \lambda_{ij}^{k\to k^{''}} +\mu_{ijd}^{k}+\mu_{ijl}^{nat.}\left( t \right)-\nu\left( t \right) \right)T^{k}$$

$$\frac{\partial Q^{k}}{\partial t}+\frac{\partial Q^{k}}{\partial a}+ \frac{\partial Q^{k}}{\partial d}=\bar{\zeta}_{g}^{k}\phi_{g}T^{k}+\sum_{k^{'}\to k} \lambda_{ij}^{k^{'}\to k}Q^{k^{'}}-\left( \sum_{k\to k^{''}} \lambda_{ij}^{k\to k^{''}} +\mu_{ijd}^{k}+\mu_{ijl}^{nat.}\left( t \right)-\nu\left( t \right) \right)Q^{k}$$

$$\frac{\partial C^{k}}{\partial t}+\frac{\partial C^{k}}{\partial a}+ \frac{\partial C^{k}}{\partial d}=\zeta_{g}^{k}\phi_{g}T^{k}+\lambda_{i}^{clear}A^{k}+\sum_{k^{'}\to k} \rho_{ij}^{k^{'}\to k}C^{k^{'}}-\left( \Lambda_{il}\left( t \right)+\sum_{k\to k^{''}} \rho_{ij}^{k\to k^{''}} +\alpha^{k}\mu_{ijd}^{k}+\mu_{ijl}^{nat.}\left( t \right)-\nu\left( t \right) \right)C^{k}$$

The following shorthand notations are used: $i,j,l,g,d$ indices dropped from state vectors for ease of reading, so $A$ stands for $A_{ijlgd}$ and so forth; $\sum_{k^{'}\to k} \lambda_{ij}^{k^{'}\to k}$ implies a sum over all the disease stages $k^{'}$ that can move into stage $k$; $\sum_{k\to k^{''}} \lambda_{ij}^{k\to k^{''}}$ is a sum over all disease stages $k^{''}$ that can be moved into from stage $k$; $\mu_{ijd}^{k}$ refers to the excess mortality at disease stage $k$ – this is zero for the majority of compartments for which there is no excess mortality due to HCV infection; the rate of change of a compartment with respect to duration, $\partial U^{k}/\partial d$ and so on, is zero for all disease stages except $k=$ DC/HCC since only in these stages is duration of the condition monitored; $\delta_{k,0}=0$ for $k\neq0$ and $\delta_{k,0}=1$ for $k=0$.

**Table 4 - Full list of parameter symbols and derived quantities with values and prior distributions.** Ranges indicate the quantity is varied in calibration or sensitivity analysis using a uniform prior distribution; single values indicated the quantity is fixed, except where an explicit prior distribution is specified.

| **Parameter** | **Description** | **Value(s) or relation to other parameters** | **Justification and references** |
| --- | --- | --- | --- |
| *Demographic parameters* |  |  |  |
| $b(t)$ | Number of births | Varies with time | Local data |
| $\mu_{ijl}^{nat.}\left( t \right)$ | Background mortality rate | $\sigma_{l}\mu_{ij}^{nat.}\left( t \right)$ | See below |
| $\sigma_{l}$ | Standardised mortality ratio | $l=1$: 5-16 | PWID SMR range from UNODC World Drug Report (2017)^15^ and Mathers et al. (2013)^16^ |
|  |  | Otherwise: 1 | Non-PWID have mortality rate given by background rate |
| $\mu_{ij}^{nat.}\left( t \right)$ | Background mortality rate | Depends on age, sex and time | Local data |
| $\nu\left( t \right)$ | Migration | Assumed equal to China | UN data |
| $\pi^{PWID}$, $\pi_{female}^{PWID}$, $\pi_{opioid-dept.}^{PWID}$ | Proportion PWID | Listed above | Local data |
|  | Proportion PWID women |  |  |
|  | Proportion PWID opioid-dependent |  |  |
| *Transmission parameters* |  |  |  |
| $p^{inf.}$ | Probability of perinatal infection | 0·027 | Ferrero et al. (2003) ^17^ |
| $\Lambda_{il}(t)$ | Force of infection; dependent on age – $i$ - and risk group – $l =$GP, PWID | GP$: \beta_{i, GP}\left( t \right) p_{all}^{eff}(t)$ | See below |
|  |  | PWID: $\beta_{i, GP}\left( t \right) p_{all}^{eff}\left( t \right)+\beta_{PWID}\left( t \right) p_{PWID}(t)$ |  |
| $p_{all}^{eff}(t)$ | Effective HCV prevalence; $I_{i}(t)$is number infected by age, $N_{i}(t)$ is population size by age | $\sum_{i} \beta_{i, GP}\left( t \right)I_{i}\left( t \right)/\sum_{i} \beta_{i, GP}\left( t \right)N_{i}(t)$ | Anderson et al. (1992)^18^ |
| $p_{PWID}(t)$ | HCV prevalence among PWID; similar prescriptions hold for MSM and FSW | $I^{pwid}(t)/N^{pwid}\left( t \right)$ | Anderson et al. (1992)^18^ |
| $\beta_{i, GP}\left( t \right)$ | Time and age dependent risk of infection in general population | $\gamma_{i}\beta_{GP}\left( t \right)$ | See below |
| $\beta_{GP}\left( t \right)$ | Time-varying contribution to general population risk of infection | Interpolated spline with risk values at years 1930, 1950, 1970, 1990 and 2005: $\xi_{1930}^{GP}$, $\xi_{1950}^{GP}$, $\xi_{1970}^{GP}$, $\xi_{1990}^{GP}$, $\xi_{2005}^{GP}$ | See below |
| $\gamma_{i}$ | Age-dependent general population risk multiplier | Interpolated spline with multiplicative values at ages 33, 66, 100 years old: $\xi_{33}^{GP}$, $\xi_{66}^{GP}$, $\xi_{100}^{GP}$ | See below |
| $\beta_{PWID}\left( t \right)$ | Risk of infection in PWID | Interpolated spline with risk values at years 1950, 1980: $\xi_{1950}^{PWID}$, $\xi_{1980}^{PWID}$ | See below |
| $\xi_{i/t}^{l}$ | Values of spline knots by risk group $l$, at time $t$ or age $i$ | $\xi_{1930}^{GP}$: $\geq$0, exponential prior (rate = 1·00) | Pre-1930 risk of infection low so greater prior weight at lower values; risks at other times drawn from effectively uninformative prior distributions; age risks completely uninformative |
|  |  | $\xi_{1950}^{GP}$: $\geq$0, exponential prior (rate = 0·05) |  |
|  |  | $\xi_{1970}^{GP}$: $\geq$0, exponential prior (rate = 0·05) |  |
|  |  | $\xi_{1990}^{GP}$: $\geq$0, exponential prior (rate = 0·05) |  |
|  |  | $\xi_{2005}^{GP}=q^{country}\xi_{1990}^{GP}$ |  |
|  |  | $\xi_{1950}^{PWID}$: $\geq$0, exponential prior (rate = 1·00) |  |
|  |  | $\xi_{1980}^{PWID}$: $\geq$0, exponential prior (rate = 0·05) |  |
|  |  | $\xi_{33}^{GP}:$0 – 1 |  |
|  |  | $\xi_{66}^{GP}$: 0 – 1 |  |
|  |  | $\xi_{100}^{GP}$: 0 – 1 |  |
| $q^{Yunnan}$ | Reduction in GP risk after 1990, | Otherwise – $q^{country}\geq0$, exponential prior (rate=5) | Assumption |
| *Natural history model parameters* |  |  |  |
| $\lambda_{i}^{clear}$ | Rate of spontaneous clearance | $p^{clear}r^{clear/chronic}$ | See below |
| $\lambda_{i}^{chronic}$ | Rate of progression to chronic disease | $\left( 1-p_{i}^{clear} \right) r^{clear/chronic}$ | See below |
| $p_{i}^{clear}$ | Proportion clearing HCV | $i\geq15$ years old: 0·25 | Grebely et al. (2014)^19^  Micallef et al. (2006)^20^ |
|  |  | $i<15$ years old: 0·55 | Vogt et al. (1999)^21^ |
| $r^{clear/chronic}$ | Rate of moving from acute to either chronic disease or susceptible | 2·18 years ^-1^ | Grebely et al. (2014)^19^ |
| $\lambda_{ij}^{0\to1}$, $\lambda_{ij}^{1\to2}$, $\lambda_{ij}^{2\to3}$, $\lambda_{ij}^{3\to4}$, $\lambda_{ij}^{3\to HCC}$, $\lambda_{ij}^{4\to HCC}$, $\lambda_{ij}^{DC\to HCC}$, $\lambda_{ij}^{4\to DC}$, $\mu_{ij}^{4}$, $\mu_{ijd}^{DC}$, $\mu_{ijd}^{HCC}$ | Age and sex dependent fibrosis progression rates and end stage disease progression rates | Calibrated; determined by age via $h_{age}^{k}$, sex via $h_{sex}^{k}$ and annual transition probabilities $f^{k\to k+1}$ where $k$denotes disease stage | See original paper for full details^1^ |
| $f_{l/u}^{0\to1}$  $f_{l/u}^{1\to2}$  $f_{l/u}^{2\to3}$  $f_{l/u}^{3\to4}$ | Annual transition probability | lower = 0·05, upper = 0·12 | Thein et al. (2008)^22^ |
|  |  | lower = 0·05, upper = 0·07 |  |
|  |  | lower = 0·08, upper = 0·13 |  |
|  |  | lower = 0·05, upper = 0·13 |  |
| $\alpha^{fibr.}$ | Scalar controlling rate of fibrosis progression | 0-1: 0 gives lower value of all fibrosis progression probabilities in above row; 1 gives upper values | Full range of values |
| $f^{3\to HCC}$ | Annual transition probability: $F_{3}$ to HCC | 0-0·02 | Dienstag et al. (2011)^23^ |
| $f^{4\to HCC}$ | Annual transition probability: $F_{4}$ to HCC | 0·01-0·09 | Fattovich et al. (1997)^24^  Kato et al. (1994)^25^ |
| $f^{DC\to HCC}$ | Annual transition probability: DC to HCC | 0·03-0·10 | Planas et al. (2004)^26^ |
| $f^{4\to DC}$ | Annual transition probability: $F_{4}$ to DC | 0·02-0·06 | Fattovich et al. (1997)^24^ |
| $f^{4\to\mu}$ | Annual probability of mortality from $F_{4}$ | 0·02-0·04 | D’Amico et al. (2006)^27^ |
| $f^{DC\to\mu}$ | Annual probability of mortality from DC | 1^st^ year: 0·07-0·25 | Fattovich et al. (1997)^24^ |
|  |  | Later years: 0·07-0·18 |  |
| $f^{HCC\to\mu}$ | Annual probability of mortality from HCC | 1^st^ year: 0·53-0·75 | Altekruse et al. (2009)^28^  Shiratori et al. (1995)^29^ |
|  |  | Later years: 0·09-0·38 |  |
| $h_{age}^{fibr.}$, $h_{age}^{j\to HCC}$, $h_{age}^{4\to DC},$ $h_{age}^{cirr.\to\mu}$, $h_{age}^{HCC\to\mu}$ | Proportional difference 70- to 20-year-old disease progression rates | 1-10 | Sweeting et al. (2006)^30^ |
| $h_{sex}^{fibr.}$, $h_{sex}^{3\to HCC}$, $h_{sex}^{4\to HCC},$ $h_{sex}^{DC\to HCC},$ $h_{sex}^{4\to DC},$  $h_{sex}^{cirr.\to\mu}$,$h_{sex}^{HCC\to\mu}$ | Proportional difference male to female disease progression rates | 1-2 | Sweeting et al. (2006)^30^ |
| *Treatment, prevention and intervention parameters* |  |  |  |
| $\zeta_{g}^{k}$ (PEG-IFN + RBV) | Proportion achieving SVR by genotype g (no treatment DC or HCC) | $\zeta_{1}^{k}$ = 0·44 | Yee et al. (2015)^31^ |
|  |  | $\zeta_{2}^{k}$ = 0·73 | Yee et al. (2015)^31^ |
|  |  | $\zeta_{3}^{k}$ = 0·73 | Yee et al. (2015)^31^ |
|  |  | $\zeta_{4}^{k}$ = 0·53 | Yee et al. (2015)^31^ |
|  |  | $\zeta_{5}^{k}$ = 0·73 | Yee et al. (2015)^31^  Nguyen et al. (2005)^32^ |
|  |  | $\zeta_{6}^{k}$ = 0·75 | Bunchorntavakul (2013)^33^ |
| $\zeta_{g}^{k}$ (DAAs) | Proportion achieving SVR under DAAs by disease stage (no treatment HCC) | $F_{0}$ to $F_{4}$: 0·98 | Forns et al. (2017)^34^ - lower confidence interval to be conservative; genotype 3 assumed equal to others |
|  |  | DC: 0·85 | Lens et al. (2017)^35^ |
| $\bar{\zeta}_{g}^{k}$ | Proportion not achieving SVR | $\bar{\zeta}_{g}^{k}=1-\zeta_{g}^{k}$ | Definition |
| $\phi_{g}$ (PEG-IFN + RBV) | Mean rate of treatment course by genotype $g$ | $\phi_{1}=$ 1/48 weeks | Strader et al. (2004)^36^  WHO (2014)^37^ |
|  |  | $\phi_{2}=$ 1/24 weeks | Strader et al. (2004)^36^  WHO (2014)^37^ |
|  |  | $\phi_{3}=$ 1/24 weeks | Strader et al. (2004)^36^  WHO (2014)^37^ |
|  |  | $\phi_{4}=$ 1/48 weeks | WHO (2014)^37^ |
|  |  | $\phi_{5}=$ 1/24 weeks | Nguyen et al. (2005)^32^  Strader et al. (2004)^36^ |
|  |  | $\phi_{6}=$ 1/48 weeks | Nguyen et al. (2005)^32^  Strader et al. (2004)^36^ |
| $\phi_{g}$ (DAAs) | Mean rate of treatment | F^0^ to F^4^: $\phi_{g}$ = 1/12 weeks | Forns et al. (2017)^34^ |
|  |  | DC: $\phi_{g}$ = 1/24 weeks | Lens et al. (2017)^35^ |
| $a_{min}$ (PEG-IFN + RBV) | Minimum age of treatment | 15 years old | Approximate minimum age used in guidelines |
| $a_{min}$ (DAAs) | Minimum age of treatment | 0 years old | Assume ongoing clinical trials will lead to all-age treatment, see Ohmer et al. (2016)^38^ |
| $\delta_{delay}^{PWID re-treat}$ | Average to possible retreatment in PWID after cure | 5 years (varied in one way SA: 1-5 years) | Likelihood of multiple retreatments in PWID has not been established; we assume a 5 year average waiting time before retreatment after reinfection |
| $\theta_{reinf}$ | Relative risk of reinfection compared to primary infection | 1 (varied in one-way SA: 0·2-1) | Evidence on relative risk of reinfection mixed;^39–44^ conservative value of one chosen (conservative as increases incidence so worsens outcomes) |
| $\rho_{ij}^{0\to1}$, $\rho_{ij}^{1\to2}$, $\rho_{ij}^{2\to3}$, $\rho_{ij}^{3\to4}$, $\rho_{ij}^{3\to HCC}$, $\rho_{ij}^{4\to HCC}$, $\rho_{ij}^{DC\to HCC}$, $\rho_{ij}^{4\to DC}$ | Age and sex dependent fibrosis progression rates after achieving SVR | $\rho_{ij}^{k\to k+1}=\alpha^{k\to k+1}\lambda_{ij}^{k\to k+1}$ | See below |
| $\alpha^{0\to1}$, $\alpha^{1\to2}$, $\alpha^{2\to3}$, $\alpha^{3\to4}$, $\alpha^{3\to HCC}$ | Hazard ratio SVR vs. non-SVR: progression rates before compensated cirrhosis | 0 | George et al. (2009)^45^ and Lee et al. (2014)^46^ – as a conservative measure no regression is modelled |
| $\alpha^{4\to HCC}$ | Hazard ratio SVR vs. non-SVR: HCC rate | 0·29 | Nahon et al. (2017)^47^ |
| $\alpha^{DC\to HCC}$ | Hazard ratio SVR vs. non-SVR: HCC rate from DC | 0·33 | Cheung et al. (2016)^48^ |
| $\alpha^{4\to DC}$ | Hazard ratio SVR vs. non-SVR: DC rate | 0·26 | Nahon et al. (2017)^47^ |
| $\alpha^{4}$, $\alpha^{DC}$, $\alpha^{HCC}$ | Hazard ratio SVR vs. non-SVR: mortality rates | $\alpha^{4}$ = 0 | Bruno et al. (2016)^49^ |
|  |  | $\alpha^{DC}$ = 1 | Cheung et al. (2016)^48^ |
|  |  | $\alpha^{HCC\to\mu}$ = 1 (SVR after development of HCC – i.e. no treatment effect so treatment not modelled after onset of HCC) | Pol et al. (2016)^50^ |
|  |  | $\alpha^{HCC}$ = 0·41 (SVR before development of HCC) | Bruno et al. (2017)^51^ |
| $\delta^{k}$ | Diagnosis rates | Varies with intervention | See below |
|  |  | Varies with intervention |  |
| $\omega^{k}$ | Relative probability of diagnosis by stage $k$, without outreach screening | $\omega^{0}=$ 0·01/4 | Assume majority of people come for diagnosis at the end stages of disease |
|  |  | $\omega^{1}=$ 0·01/4 |  |
|  |  | $\omega^{2}=$0·01/4 |  |
|  |  | $\omega^{3}=$0·01/4 |  |
|  |  | $\omega^{4}=$ 0·1 |  |
|  |  | $\omega^{DC}=$ 0·4 |  |
|  |  | $\omega^{HCC}=$0·4 |  |
| $\Delta$ | Diagnosis rate | Varies with intervention | Not applicable |
| $\tau(t)$ | Treatment rate | Varies with intervention | Not applicable |
| $\tau^{DC}\left( t \right)$, $\tau^{HCC}\left( t \right)$ | Treatment rate in DC and HCC | DC: no treatment under PEG-IFN as contraindicated. Under DAAs, treatment rates as for $\tau(t)$, see above  HCC: impact of SVR on patients with HCC under any treatment is contested so zero treatment in this group | Gambato et al. (2014)^52^  Pol et al. (2016)^50^ |
| $\Omega_{GP risk red.}^{intervention}$ | Reduction in GP risk in blood and infection control intervention | Varies with time | Local data on intervention coverage |
| $\Omega_{PWID harm red.}$ | Reduction in PWID risk in those covered by combination NSP and OST | 75% | Hagan et al. (2011)^53^ |
| $\kappa_{PWID harm red.}^{historical}$ | Coverage of historical harm reduction interventions | Varies with time | Local data on intervention coverage |
| $\kappa_{PWID harm red.}^{intervention}$ | Coverage of NSP + OST combination in PWID harm reduction intervention | Varies with time | Local data on intervention coverage |

**Table 5 - List of calibrated parameters with prior distributions.**

| **Parameter** | **Description** | **Prior distribution** | **Values and details** |
| --- | --- | --- | --- |
| $\pi^{PWID}$ | Proportion PWID | Uniform | Range of values informed by width of uncertainty reported for China as a whole, see Degenhardt et al. (2017)^7^ |
| $\sigma_{l}$ | Standardised mortality ratio among PWID | Uniform | 5-16^15,16^ |
| $\xi_{1930}^{GP}$ | Values of spline knots by risk group $l$, at time $t$ or age $i$; FSW and MSM risk groups follow same prescription as PWID detailed here. | Exponential | Rate = 1·00 |
| $\xi_{1950}^{GP}$ |  | Exponential | Rate = 0·05 |
| $\xi_{1970}^{GP}$ |  | Exponential | Rate = 0·05 |
| $\xi_{1990}^{GP}$ |  | Exponential | Rate = 0·05 |
| $\xi_{2005}^{GP}$ |  | $q^{country}\xi_{1990}^{GP}$ | NA: $q^{Yunnan}$ distribution given below |
| $\xi_{1950}^{PWID}$ |  | Exponential | Rate = 1·00 |
| $\xi_{1980}^{PWID}$ |  | Exponential | Rate = 0·05 |
| $\xi_{33}^{GP}$ |  | Uniform | 0-1 |
| $\xi_{66}^{GP}$ |  | Uniform | 0-1 |
| $\xi_{100}^{GP}$ |  | Uniform | 0-1 |
| $q^{country}$ | Reduction in GP risk after 1990 | Otherwise: Exponential | Rate = 5 |
| $\alpha^{fibr.}$ | Scalar controlling rate of fibrosis progression | Uniform | 0-1 (0 gives lower value of all fibrosis progression probabilities; 1 gives upper values^22^) |
| $f^{3\to HCC}$ | Annual transition probability: $F_{3}$ to HCC | Uniform | 0-0·02^23^ |
| $f^{4\to HCC}$ | Annual transition probability: $F_{4}$ to HCC | Uniform | 0·01-0·09^24,25^ |
| $f^{DC\to HCC}$ | Annual transition probability: DC to HCC | Uniform | 0·03-0·10^26^ |
| $f^{4\to DC}$ | Annual transition probability: $F_{4}$ to DC | Uniform | 0·02-0·06^24^ |
| $f^{4\to\mu}$ | Annual probability of mortality from $F_{4}$ | Uniform | 0·02-0·04^27^ |
| $f^{DC\to\mu}$, 1^st^ year | Annual probability of mortality from DC | Uniform | 0·07-0·25^24^ |
| $f^{DC\to\mu}$, later years |  | Uniform | 0·07-0·18^24^ |
| $f^{HCC\to\mu}$, 1^st^ year | Annual probability of mortality from HCC | Uniform | 0·53-0·75^28,29^ |
| $f^{HCC\to\mu}$, later years |  | Uniform | 0·09-0·38^28,29^ |
| $h_{age}^{fibr.}$, $h_{age}^{j\to HCC}$, $h_{age}^{j\to DC},$ $h_{age}^{cirr.\to\mu}$, $h_{age}^{HCC\to\mu}$ | Proportional difference 70- to 20-year-old disease progression rates | Uniform | 1-10^30^ |
| $h_{sex}^{fibr.}$, $h_{sex}^{j\to HCC},$ $h_{sex}^{j\to DC},h_{sex}^{cirr.\to\mu}$,$h_{sex}^{HCC\to\mu}$ | Proportional difference male to female disease progression rates | Uniform | 1-2^30^ |

## Supplementary results tables

**Table 6 -** **Cost-effectiveness analysis results.** ICER = incremental cost-effectiveness ratio. USD = United States Dollars. DALYs = disability-adjusted life years. PWID = people who inject drugs. MSM = men who have sex with men. DAAs = direct-acting antivirals. FSW = female sex workers. Shown are incremental results comparing the incremental costs, DALYs averted, and ICERs between each successively added element (order is determined by order of mean ICER); the first intervention – screen PWID – is compared to a scenario in which DAAs are introduced (scenario A). DALYs averted and cost increases are presented as mean net-present values (according to a discount rate of 3% per year). All results are based on lifetime horizons (to 2100). The ‘additional annual investment’ column is the net-present value of the total investment spend on the intervention (treatment and screening, see Figure 2) from 2019 to 2030 less the equivalent amount for the preceding scenario (converted to an annual figure). For reference, the total annual HCV investment spend in the baseline (PEG-IFN+RBV) is 7.0 million USD, while in the + DAAs intervention (scenario A) it is 8.1 million USD (a difference of 1.18 million USD). Numbers converted to 2019 USD according to an exchange rate of 1 USD = 6.75 Chinese Yuan (as per first three months of 2019).^54^

| **Scenario added** | **Mean ICER (USD per DALY averted)** | **Cost increase on previous scenario (millions USD)** | **Additional DALYs averted (thousands)** | **Additional annual investment in intervention treatment and screening (millions USD)** |
| --- | --- | --- | --- | --- |
| E. Screen PWID | 1,003 | 14.71 | 14·67 | 2.23 |
| F. Screen in HIV care | 1,116 | 40.42 | 36·21 | 5.64 |
| C. Screen MSM | 1,743 | 0.54 | 0·31 | 0.07 |
| B. DAAs for majority newly diagnosed | 1,923 | 169.61 | 88·2 | 23.48 |
| H. Screen 40 year olds | 1,982 | 5.59 | 2·82 | 0.60 |
| D. Screen FSW | 5,466 | 0.47 | 0·09 | 0.09 |
| G. Screen pregnant women | 11,756 | 16.01 | 1·36 | 1.58 |

**Supplementary results figures**

**Figure 1 - Sensitivity of mean incremental cost-effectiveness ratio in scenario A (+ direct-acting antivirals) to changes in methodological parameters.** Vertical black line indicates mean ICER in primary analysis. The order of values in the y-axis labels indicates the direction in which the value is changed (for instance, the leftmost ICER plotted against discount rate corresponds to a discount rate of 0% and so on). DALY = disability-adjusted life year. ESLD = end-stage liver disease. ICER = Incremental cost-effectiveness ratio. USD = United States Dollars.

**Figure 2 - Variance of mean incremental cost-effectiveness ratio under scenario A (+ direct-acting antivirals) attributed to each cost parameter.** A positive result indicates that an increase in the cost component results in an increase in mean ICER: for example, increasing DAA costs relative to PEG-IFN+RBV costs results in reduced cost effectiveness (higher ICER). Conversely, negative results mean increasing the cost component decreases the ICER: for example, increasing the cost of care results in lower ICERs. DAA = direct-acting antivirals. PEG-IFN+RBV = pegylated interferon + ribavirin. SVR = sustained viral response. HCV = hepatitis C virus.

**Figure 3 – Cost-effectiveness acceptability frontier.** Dashed, coloured lines indicate the proportion of all runs that are cost-effective at a particular cost-effectiveness threshold for each intervention element (interventions are added sequentially in order of mean ICER; intervention E is added to a baseline scenario of status quo + DAAs as in the analysis to answer research question 2); the dashed lines are, therefore, cost-effectiveness acceptability curves for each intervention. The intervention with the highest expected net benefit is deemed the optimal intervention (provided it is positive, i.e. cost-effective at that threshold): this is indicated as solid, coloured lines on the figure. Vertical dashed lines are the break points where one optimal intervention is superseded by another – that is, an additional intervention component is added to the optimal package; the values of the dashed vertical lines therefore correspond to the ICERs of each intervention element as it is added, see Table 4. USD = United States Dollars. PWID = people who inject drugs. MSM = men who have sex with men. DAAs = direct-acting antivirals. FSW = female sex workers

**Figure 4 - Sensitivity of mean return on investment in scenario A (+ direct-acting antivirals) to changes in methodological parameters.** Vertical black line indicates mean ROI in the primary analysis. The order of values in the y-axis labels indicates the direction in which the value is changed (for instance, the leftmost ROI plotted against discount rate corresponds to a discount rate of 12% and so on). ROI = return on investment. USD = United States Dollars.

**Figure 5 - Variance of return on investment of optimal package of interventions by economic parameters.** A positive result indicates that an increase in the cost component results in an increase in mean ROI: for example, increasing the cost of care results in higher ROI. Conversely, negative results mean increasing the cost component decreases the ROI: for example, increasing DAA costs relative to PEG-IFN+RBV costs results in reduced ROI. DAA = direct-acting antivirals. PEG-IFN+RBV = pegylated interferon + ribavirin. SVR = sustained viral response. HCV = hepatitis C virus.

**Figure 6 - Sensitivity of return on investment to changes in direct-acting antiviral and care costs.** Dashed lines indicate the central values used in the rest of the analysis. DAA = direct-acting antiviral. ROI = return on investment. USD = United States Dollars.

**Figure 7 – Projected number of deaths under status quo scenario and optimum intervention.** Optimum intervention consists of scenarios E, F, C, B, and H

**Figure 8 – Projected prevalence under status quo scenario and optimum intervention.** Optimum intervention consists of scenarios E, F, C, B, and H

**Figure 9 – Projected incidence under status quo scenario and optimum intervention.** Optimum intervention consists of scenarios E, F, C, B, and H

## References

1 Heffernan A, Cooke GS, Nayagam S, Thursz M, Hallett TB. Scaling up prevention and treatment towards the elimination of hepatitis C: a global mathematical model. *The Lancet* 2019; published online Jan. DOI:10.1016/S0140-6736(18)32277-3.

2 Raftery AE, Bao L. Estimating and projecting trends in HIV/AIDS generalized epidemics using incremental mixture importance sampling. *Biometrics* 2010; **66**: 1162–73.

3 Schroeder SA. Incidence, prevalence, and hybrid approaches to calculating disability-adjusted life years. *Popul Health Metr* 2012; **10**. DOI:10.1186/1478-7954-10-19.

4 Barton GR, Briggs AH, Fenwick EAL. Optimal cost-effectiveness decisions: the role of the cost-effectiveness acceptability curve (CEAC) the cost-effectiveness acceptability frontier (CEAF), and the expected value of perfection information (EVPI). *Value Health* 2008; **11**: 886–97.

5 Claxton K. The irrelevance of inference: a decision-making approach to the stochastic evaluation of health care technologies. *J Health Econ* 1999; **18**: 341–64.

6 Zhang G, Jiang H, Shen J, Wen P, Liu X, Hao W. Estimating prevalence of illicit drug use in Yunnan, China, 2011–15. *Front Psychiatry* 2018; **9**. DOI:10.3389/fpsyt.2018.00256.

7 Degenhardt L, Peacock A, Colledge S, *et al.* Global prevalence of injecting drug use and sociodemographic characteristics and prevalence of HIV, HBV, and HCV in people who inject drugs: a multistage systematic review. *Lancet Glob Health* 2017.

8 The Polaris Observatory HCV Collaborators. Global prevalence and genotype distribution of hepatitis C virus infection in 2015: a modelling study. *Lancet Gastroenterol Hepatol* 2017; **2**: 161–176.

9 王晓霞, 邹正升, 李保森, *et al.* 2002 至 2011 年中国 “北方” 地区住院肝硬化患者病因构成及变化趋势分析. 实用肝脏病杂志 2014; **17**: 154–8.

10 吴安燕, 邓世康, 杨晋辉. 1545 例肝硬化患者病因分析. 云南医药 2017; **38**: 109–11.

11 王付彬. 272 例肝硬化患者流行病学调查报告. 邯郸医学高等专科学校学报 2005; **18**: 18–9.

12 杨志国, 陈晓慧, 沈敏, 汪茂荣. 580 例肝硬化住院患者病因分析. 中国肝脏病杂志 电子版 2014; **6**: 47–9.

13 de Martel C, Maucort-Boulch D, Plummer M, Franceschi S. World-wide relative contribution of hepatitis B and C viruses in hepatocellular carcinoma. *Hepatology* 2015; **62**: 1190–200.

14 Zhu RX, Seto W-K, Lai C-L, Yuen M-F. Epidemiology of hepatocellular carcinoma in the Asia-Pacific region. *Gut Liver* 2016; **10**. DOI:10.5009/gnl15257.

15 United Nations Office on Drugs and Crime. Booklet 2: Global overview of drug demand and supply. In: World Drug Report 2017. Vienna, Austria: United Nations Publication, 2017. https://www.unodc.org/wdr2017/field/Booklet_2_HEALTH.pdf (accessed June 20, 2018).

16 Mathers BM, Degenhardt L, Bucello C, Lemon J, Wiessing L, Hickman M. Mortality among people who inject drugs: a systematic review and meta-analysis. *Bull World Health Organ* 2013; **91**: 102–23.

17 Ferrero S, Lungaro P, Bruzzone BM, Gotta C, Bentivoglio G, Ragni N. Prospective study of mother-to-infant transmission of hepatitis C virus: a 10-year survey (1990–2000). *Acta Obstet Gynecol Scand* 2003; **82**: 229–234.

18 Anderson RM, May RM, Anderson B. Infectious diseases of humans: dynamics and control. Wiley Online Library, 1992.

19 Grebely J, Page K, Sacks-Davis R, *et al.* The effects of female sex, viral genotype, and IL28B genotype on spontaneous clearance of acute hepatitis C virus infection. *Hepatology* 2014; **59**: 109–20.

20 Micallef JM, Kaldor JM, Dore GJ. Spontaneous viral clearance following acute hepatitis C infection: a systematic review of longitudinal studies. *J Viral Hepat* 2006; **13**: 34–41.

21 Vogt M, Lang T, Frösner G, *et al.* Prevalence and clinical outcome of hepatitis C infection in children who underwent cardiac surgery before the implementation of blood-donor screening. *N Engl J Med* 1999; **341**: 866–870.

22 Thein H-H, Yi Q, Dore GJ, Krahn MD. Estimation of stage-specific fibrosis progression rates in chronic hepatitis C virus infection: a meta-analysis and meta-regression. *Hepatology* 2008; **48**: 418–31.

23 Dienstag JL, Ghany MG, Morgan TR, *et al.* A prospective study of the rate of progression in compensated, histologically advanced chronic hepatitis C. *Hepatology* 2011; **54**: 396–405.

24 Fattovich G, Giustina G, Degos F, *et al.* Morbidity and mortality in compensated cirrhosis type C: a retrospective follow-up study of 384 patients. *Gastroenterology* 1997; **112**: 463–72.

25 Kato Y, Nakata K, Nagataki S, *et al.* Risk of hepatocellular carcinoma in patients with cirrhosis in Japan. Analysis of infectious hepatitis viruses. *Cancer* 1994; **74**: 2234–8.

26 Planas R, Ballesté B, Antonio Álvarez M, *et al.* Natural history of decompensated hepatitis C virus-related cirrhosis. A study of 200 patients. *J Hepatol* 2004; **40**: 823–30.

27 D’Amico G, Garcia-Tsao G, Pagliaro L. Natural history and prognostic indicators of survival in cirrhosis: a systematic review of 118 studies. *J Hepatol* 2006; **44**: 217–31.

28 Altekruse SF, McGlynn KA, Reichman ME. Hepatocellular carcinoma incidence, mortality, and survival trends in the United States from 1975 to 2005. *J Clin Oncol* 2009; **27**: 1485–91.

29 Shiratori Y, Shiina S, Imamura M, *et al.* Characteristic difference of hepatocellular carcinoma between hepatitis B- and C-viral infection in Japan. *Hepatology* 1995; **22**: 1027–1033.

30 Sweeting MJ, De Angelis D, Neal KR, *et al.* Estimated progression rates in three United Kingdom hepatitis C cohorts differed according to method of recruitment. *J Clin Epidemiol* 2006; **59**: 144–52.

31 Yee BE, Nguyen NH, Zhang B, *et al.* Sustained virological response and its treatment predictors in hepatitis C virus genotype 4 compared to genotypes 1, 2, and 3: a meta-analysis. *BMJ Open Gastroenterol* 2015; **2**: e000049.

32 Nguyen MH, Keeffe EB. Prevalence and treatment of hepatitis C virus genotypes 4, 5, and 6. *Clin Gastroenterol Hepatol* 2005; **3**: s97–101.

33 Bunchorntavakul C. Hepatitis C genotype 6: a concise review and response-guided therapy proposal. *World J Hepatol* 2013; **5**: 496.

34 Forns X, Lee SS, Valdes J, *et al.* Glecaprevir plus pibrentasvir for chronic hepatitis C virus genotype 1, 2, 4, 5, or 6 infection in adults with compensated cirrhosis (EXPEDITION-1): a single-arm, open-label, multicentre phase 3 trial. *Lancet Infect Dis* 2017; **17**: 1062–8.

35 Lens S, Fernández I, Rodríguez-Tajes S, *et al.* Interferon-free therapy in elderly patients with advanced liver disease. *Am J Gastroenterol* 2017; **112**: 1400.

36 Strader DB, Wright T, Thomas DL, Seeff LB. Diagnosis, management, and treatment of hepatitis C. *Hepatology* 2004; **39**: 1147–71.

37 World Health Organization. Guidelines for the screening, care and treatment of persons with hepatitis C infection. World Health Organization, 2014 http://apps.who.int/iris/bitstream/10665/111747/5/9789241548755_mon.pdf (accessed July 25, 2017).

38 Ohmer S, Honegger J. New prospects for the treatment and prevention of hepatitis C in children. *Curr Opin Pediatr* 2016; **28**: 93–100.

39 Micallef JM, Macdonald V, Jauncey M, *et al.* High incidence of hepatitis C virus reinfection within a cohort of injecting drug users. *J Viral Hepat* 2007; **14**: 413–8.

40 Aitken CK, Lewis J, Tracy SL, *et al.* High incidence of hepatitis C virus reinfection in a cohort of injecting drug users. *Hepatology* 2008; **48**: 1746–52.

41 Dore GJ, Altice F, Litwin AH, *et al.* Elbasvir–grazoprevir to treat hepatitis C virus infection in persons receiving opioid agonist therapy: a randomized trial. *Ann Intern Med* 2016; **165**: 625.

42 Mehta SH, Cox A, Hoover DR, *et al.* Protection against persistence of hepatitis C. *The Lancet* 2002; **359**: 1478–83.

43 Midgard H, Bjøro B, Mæland A, *et al.* Hepatitis C reinfection after sustained virological response. *J Hepatol* 2016; **64**: 1020–6.

44 World Hepatitis Alliance. Hepatitis C virus reinfection is uncommon after being cured with DAAs. 2017; published online May 22. http://www.worldhepatitisalliance.org/latest-news/infohep/3140568/hepatitis-c-virus-reinfection-uncommon-after-being-cured-daas (accessed Aug 2, 2018).

45 George SL, Bacon BR, Brunt EM, Mihindukulasuriya KL, Hoffmann J, Di Bisceglie AM. Clinical, virologic, histologic, and biochemical outcomes after successful HCV therapy: a 5-year follow-up of 150 patients. *Hepatology* 2009; **49**: 729–38.

46 Lee YA, Friedman SL. Reversal, maintenance or progression: what happens to the liver after a virologic cure of hepatitis C? *Antiviral Res* 2014; **107**: 23–30.

47 Nahon P, Bourcier V, Layese R, *et al.* Eradication of hepatitis C virus infection in patients with cirrhosis reduces risk of liver and non-liver complications. *Gastroenterology* 2017; **152**: 142–156.

48 Cheung MCM, Walker AJ, Hudson BE, *et al.* Outcomes after successful direct acting antiviral therapy for patients with chronic hepatitis C and decompensated cirrhosis. *J Hepatol* 2016; published online July. DOI:10.1016/j.jhep.2016.06.019.

49 Bruno S, Di Marco V, Iavarone M, *et al.* Survival of patients with HCV cirrhosis and sustained virologic response is similar to the general population. *J Hepatol* 2016; **64**: 1217–23.

50 Pol S. Lack of evidence of an effect of direct acting antivirals on the recurrence of hepatocellular carcinoma. *J Hepatol* 2016. http://www.natap.org/2016/HCV/PIIS0168827816302598.pdf (accessed Oct 13, 2017).

51 Bruno S, Di Marco V, Iavarone M, *et al.* Improved survival of patients with hepatocellular carcinoma and compensated hepatitis C virus-related cirrhosis who attained sustained virological response. *Liver Int* 2017; **37**: 1526–34.

52 Gambato M, Lens S, Navasa M, Forns X. Treatment options in patients with decompensated cirrhosis, pre-and post-transplantation. *J Hepatol* 2014; **61**: S120–31.

53 Hagan H, Pouget ER, Des Jarlais DC. A systematic review and meta-analysis of interventions to prevent hepatitis C virus infection in people who inject drugs. *J Infect Dis* 2011; **204**: 74–83.

54 ofx.com. Yearly Average Rates. https://www.ofx.com/en-au/forex-news/historical-exchange-rates/yearly-average-rates/ (accessed July 30, 2019).
